# The selective cathepsin K inhibitor MIV-711 attenuates joint pathology in experimental animal models of osteoarthritis

**DOI:** 10.1186/s12967-018-1425-7

**Published:** 2018-03-09

**Authors:** Erik Lindström, Biljana Rizoska, Karin Tunblad, Charlotte Edenius, Alison M. Bendele, Don Maul, Michael Larson, Neha Shah, Valerie Yoder Otto, Chris Jerome, Urszula Grabowska

**Affiliations:** 1 Medivir AB, Box 1086, 141 22 Huddinge, Sweden; 2 Bolder BioPATH Inc, Boulder, CO USA; 3 PCRS Inc, Fort Collins, CO USA; 4 Ibex Preclinical Research Inc, Logan, UT USA; 5 Numira Inc, Salt Lake City, UT USA; 6 Think Bone Consulting, Langley, WA USA

**Keywords:** Cathepsin K, Osteoarthritis, HP-1, CTX-I, CTX-II, Subchondral bone, Cartilage

## Abstract

**Background:**

MIV-711 is a highly potent and selective cathepsin K inhibitor. The current article summarizes the therapeutic effects of MIV-711 on joint pathology in rabbits subjected to anterior cruciate ligament transection (ACLT), and the prophylactic effects on joint pathology in dogs subjected to partial medial meniscectomy, two surgical models of osteoarthritis (OA).

**Methods:**

Starting 1 week after surgery, rabbits were dosed daily via oral gavage with either MIV-711 or vehicle (n = 7/group) for 7 weeks. The four treatment groups were: (1) sham + vehicle; (2) ACLT + vehicle; (3) ACLT + MIV-711, 30 µmol/kg and (4) ACLT + MIV-711, 100 µmol/kg. Subchondral bone and articular cartilage structures were assessed by µCT, histomorphometry, and scoring. Dogs subjected to partial medial meniscectomy received either MIV-711 (30 µmol/kg) or vehicle (n = 15/group) via oral gavage once daily, starting 1 day before meniscectomy, for 28 days. Cartilage degradation was assessed at the macroscopic and microscopic levels. The exposures of MIV-711 were assessed in both studies and biomarkers reflecting bone resorption (HP-1 in rabbits, CTX-I in dogs) and cartilage degradation (CTX-II) were measured.

**Results:**

In ACLT rabbits, MIV-711 decreased HP-1 levels by up to 72% (*p *< 0.001) and CTX-II levels by up to 74% (*p *< 0.001) compared to ACLT vehicle controls. ACLT surgery significantly reduced the total thickness of the subchondral bone plate and reduced trabecular bone volume in the femur and tibia. These effects were reversed by MIV-711. ACLT resulted in cartilage thickening, which was attenuated by MIV-711. MIV-711 did not affect osteophyte formation or Mankin scores. In dogs, MIV-711 reduced CTX-I and CTX-II levels by 86% (*p *< 0.001) and 80% (*p *< 0.001), respectively. Synovial CTX-II levels were reduced by 55–57% (*p *< 0.001) compared to baseline. MIV-711-treated animals had 25–37% lower macroscopic scores in the femur condyles and 13–33% lower macroscopic scores in the tibial plateaus.

**Conclusions:**

MIV-711 prevents subchondral bone loss and partially attenuates cartilage pathology in two animal models of OA. These beneficial effects of MIV-711 on joint pathology are observed in conjunction with decreases in bone and cartilage biomarkers that have been shown to be clinically attainable in human. The data support the further development of MIV-711 for the treatment of OA.

**Electronic supplementary material:**

The online version of this article (10.1186/s12967-018-1425-7) contains supplementary material, which is available to authorized users.

## Background

Osteoarthritis (OA) is a common musculoskeletal disease characterized by progressive structural damage to the joint leading to symptoms such as joint stiffness and pain [1]. OA affects the whole joint and involves both cartilage and subchondral bone degeneration, and synovial inflammation. While the disease is characterized by cartilage degradation, the role of subchondral bone in the development and progression of OA has received increasing recognition in recent years [2–4]. A high degree of subchondral bone turnover was shown to predict subsequent joint space narrowing in OA patients using scintigraphy [5] and increased subchondral bone turnover in OA patients was demonstrated using biomarkers [6]. Bone attrition, which reflects subchondral bone loss and flattening, probably due to inadequate bone quality, is associated with bone marrow lesions [7], cartilage loss [8] and structural abnormalities such as malalignment [9]. Alterations in subchondral bone turnover seem to occur early in OA and may precede cartilage lesions [10]. However, it is also well recognized that there is extensive cross-talk between subchondral bone and articular cartilage and early biomechanical changes in one compartment are likely to affect the other [11]. Progress has been made in understanding the molecular signaling mechanisms between these two compartments, but they are not yet completely understood. Importantly, it has been shown in clinical trials that agents that inhibit bone resorption such as strontium ranelate [12], risedronate [13] and calcitonin [14] also show positive effects on OA-related endpoints such as joint space narrowing and on patient reported outcomes such as WOMAC scores in Phase II studies. However, either inconsistent efficacy or safety concerns have precluded approval of these agents for OA.

Cathepsin K is predominately expressed in osteoclasts and is a key enzyme involved in bone resorption through cleavage of type I collagen [15]. Cathepsin K is also expressed in chondrocytes in cartilage, where it can cleave type II collagen and aggrecan, the main components of the cartilage matrix [16–18]. Patients with pycnodysostosis, a disorder caused by inactivating mutations in the cathepsin K gene, exhibit abnormally dense bone (osteopetrosis), and this effect is reproduced in transgenic mice that are deficient in cathepsin K [19, 20]. Selective cathepsin K inhibitors increase bone mineral density in ovariectomized monkeys [21, 22] and clinically in post-menopausal women with osteoporosis [23, 24]. Furthermore, the most advanced cathepsin K inhibitor, odanacatib, reduced the incidence of vertebral and hip fractures in postmenopausal women in a Phase III study [25].

While numerous studies have demonstrated that cathepsin K inhibitors can provide beneficial effects on osteoporotic bone, less is known about the role of cathepsin K on subchondral bone and articular cartilage. Available data in preclinical models of joint degeneration support an active role for cathepsin K in the disease process, as demonstrated in studies using experimental cathepsin K inhibitors. Connor et al. [26] showed that the cathepsin K inhibitor SB553484, albeit with poor selectivity versus other cathepsins, exerted cartilage protection in dogs subjected to partial medial meniscectomy. Hayami et al. [27] demonstrated that the selective cathepsin K inhibitor L-006235 reversed subchondral bone loss in rabbits subjected to anterior cruciate ligament transection (ACLT) and that the subchondral bone protection was associated with some protection of cartilage. However, neither of these two compounds has progressed into clinical development. Thus, it is difficult to interpret if the doses used, the exposures reached and the effects seen in the preclinical models bear any relevance for a potential therapeutic effect in clinical OA.

MIV-711 is a highly potent and selective cathepsin K inhibitor [28] currently in Phase II for OA. The results from the initial Phase IIa study were recently reported [29]. Knee OA patients receiving once daily treatment with MIV-711 for 6 months demonstrated benefit on joint structure, with significantly lower increases in bone area and cartilage thinning in the diseased knee, as assessed by magnetic resonance imaging (MRI), compared to patients who received placebo. The current article summarizes the effects of MIV-711 on joint pathology in rabbits subjected to ACLT and dogs subjected to partial medial meniscectomy, two surgical models of OA. The doses of MIV-711 used in the animal models were aimed to produce clinically relevant exposures that are known to be safe, have been shown to engage cathepsin K in healthy volunteers and post-menopausal women and are intended to be reached in clinical studies in OA patients.

## Methods

### ACLT in rabbits

#### Animals and surgery

The animals were acquired following review and approval by the Institutional Animal Care and Use Committee (IACUC) at Numira (Salt Lake City, UT). The protocol was also reviewed and approved by the IACUC at Ibex Preclinical Research Inc. (Logan, UT). Male, New Zealand White rabbits (n = 32) were purchased from the Western Oregon Rabbit Co. (Philomath, OR). The animals were approximately 9 months old, weighed 3.7–4.5 kg at the start of the experiment and were randomized into four treatment groups based on body weight.

Pre-operative butorphanol (0.5–1.0 mg/kg, i.m.) for analgesia and glycopyrrolate (~ 0.1 mg/kg i.m.) were administered up to 15 min prior to induction. Anesthesia was induced by a mixture of ketamine (20–40 mg/kg, i.m.) and xylazine (5–10 mg/kg, i.m.), followed by placement of an endotracheal tube. General anesthesia was maintained with isoflurane. Perioperative cefazolin (40 mg/kg, i.v.) was administered, and yobine (0.2 mg/kg, i.v.) was administered to reverse the effects of xylazine after endotracheal intubation was complete. A fentanyl patch (25 µg/h) was placed on a clipped area of the skin to provide post-operative analgesia. An incision of 1.5–2.5 cm was made in the skin over the medial side of the right knee under aseptic conditions. The patella was dislocated laterally and the knee placed in full flexion. The anterior cruciate ligament (ACL) was visualized and transected with an appropriate blade. In sham animals, the ACL was visualized but not transected. The joint was irrigated with sterile saline and closed. The muscle and skin were closed with suture and/or surgical glue. Butorphanol (0.5–1 mg/kg, i.m.) was administered after extubation to provide supplemental analgesia. The animals were then individually housed in stainless steel or plastic cages.

#### Experimental design and sample collection

Starting 1 week after sham operation or ACLT, animals were dosed daily for 7 weeks by oral gavage with either MIV-711 (synthesized by Medivir and given as a suspension in 1% Methocel A4C in water) or vehicle (1% Methocel A4C in water) at a dose volume of 4 mL/kg. The four treatment groups (n = 7 in each group) were: (1) sham + vehicle; (2) ACLT + vehicle; (3) ACLT + MIV-711, 30 µmol/kg (low dose) and (4) ACLT + MIV-711, 100 µmol/kg (high dose). The doses and dosing interval were selected based on the potency of MIV-711 against rabbit cathepsin K enzyme (K_i_ = 3.3 nmol/L, [28]) and the pharmacokinetic (PK) profile of MIV-711 in normal New Zealand White rabbits. Doses selected for the rabbit ACLT study were aimed to generate exposures that were in the same range as the exposures that were well tolerated in humans and effectively reduced biomarkers of bone resorption and cartilage degradation in healthy subjects.

Twelve blood samples of 1 mL each were collected from each animal for determination of MIV-711 concentrations in a staggered manner to cover as many time points as possible. The blood samples were collected on Day 1, Day 10 and during Week 4 and Week 7. The samples were collected in EDTA coated tubes and kept on ice. The plasma was separated by centrifugation (2000*g* for 3–5 min at 4 °C) and then frozen and kept at − 70 °C until analysis.

Urine was collected in the morning from all animals for biomarker measurements before surgery, before dosing on Day 1, Day 10 and during Week 4 and Week 7. The urine was stored frozen (− 70 to − 80 °C) until analysis.

#### Micro-computed tomography (µCT)

Femora and tibias from all animals were subjected to µCT scanning without and with contrast reagent, to visualize bone and cartilage, respectively. Samples were scanned on a high-resolution, volumetric µCT scanner (μCT40, ScanCo Medical, Zurich, CH). The image data was acquired with the following parameters: 36 μm isotropic voxel resolution at 300 ms exposure time, 2000 views and 1 frame per view. Each sample was scanned twice, once for acquiring bone data and once for soft tissue data. After the bone scans, the knees were stained using hexabrix for contrast-enhanced imaging of the cartilage. The µCT-generated DICOM files were used to analyze the samples and to create volume renderings of the regions of interest (ROI). The raw data files were converted into a file format compatible with the segmentation software VHLab (Numira). VHLab was used for segmenting out the regions of interest (osteophyte, cartilage, and subchondral bone) for the femur and tibia of each sample. A region spanning 9 mm of the articulating surface of the femur and 6.5 mm of the tibia was selected for segmenting out the cartilage and subchondral bone in the anterior–posterior direction. After the segmentation process, the voxel count associated with each of the regions of interest was calculated using VHLab. The voxel count was then multiplied by the cubic voxel resolution to obtain volume measurements for the different ROI. Cartilage thickness was calculated using SCIRun (Scientific Computing and Imaging Institute, University of Utah). The distance from the bone to the outer surface of the articulating cartilage was calculated. An image was generated that translates the distances into a color map for viewing the thickness along the length of the cartilage. Similarly, the thickness and thickness map image of the subchondral bone was calculated. The trabecular bone analysis was performed on all four condyles of the knees (medial and lateral side of the femur and tibia). For the tibia, the ROI size was 3.8 × 3.8 × 1.5 mm whereas for the femur the ROI size was 2.5 × 3.0 × 2.0 mm taken from the center of each condyle.

#### Histology

Details on histology can be found in Additional file 1.

### Partial medial meniscectomy in dogs

#### Animals and surgery

The study design and animal usage were reviewed and approved by the IACUC at Preclinical Research Services (PCRS; Fort Collins, CO) for compliance with regulations before study initiation (IACUC Number 1021). Animal welfare, housing and research procedures for this study complied with the U.S. Department of Agriculture’s (USDA) Animal Welfare Act (9 CFR Parts 1, 2, and 3), the Guide for the Care and Use of Laboratory Animals, and PCRS Standard of Procedures. Thirty adult naïve female beagle dogs, with a weight of 7.5–11.3 kg and an age of approximately 6–8 months at study initiation, were used for this study. The dogs were acclimated for at least 7 days before study initiation.

At least 12 h before surgery, a fentanyl transdermal 2.5 mg patch was placed on the skin on the ventral aspect of the tail of each dog and secured into place with tape. The fentanyl patch delivered approximately 25 μg/h of analgesia for a total duration of 72–80 h. Dogs were also pre-medicated before surgery with glycopyrrolate (0.005–0.02 mg/kg), acepromazine (0.01–0.05 mg/kg) and morphine (0.5–1.0 mg/kg) injected subcutaneously. General anesthesia was induced using propofol (2–6 mg/kg) given intravenously; each dog was then intubated and maintained on isoflurane in oxygen (0.5–5%) administered to effect. The hair over the surgical site was clipped, and a preliminary aseptic prep of the area was done by alternating scrubs of chlorhexidine surgical scrub and sterile dry gauze sponges. A local anesthetic line block of lidocaine and bupivacaine (1.5 mg each) diluted in saline was administered at the incision site to provide local pre-emptive anesthesia. The limb was draped with a sterile fenestrated surgical drape. A small 2 cm medial parapatellar skin incision and a subsartorius capsular approach were made to expose the meniscus. For removal of the anterior one-half of the medial meniscus, the cranial meniscotibial ligament was incised, and the capsular attachments to the cranial pole of the meniscus were separated sharply with a scalpel under direct vision, sparing injury to the articular cartilage or the ACL. The medial meniscus was reflected medially with a hemostat. The meniscus was transected just cranial to the medial collateral ligament and removed. A full thickness cut was made through the meniscus, removing approximately 1/2 of the meniscus. The synovium and skin were closed with 3–0 Polysorb suture. Animals were monitored closely throughout the recovery and post-operative period. Animals were observed for vocalization, movement, agitation, heart rate and respiratory rate. The transdermal fentanyl patch was removed at roughly 72 h post-op. No dog developed a non-weight bearing lameness following fentanyl patch removal.

#### Experimental design and sample collection

Dogs subjected to partial medial meniscectomy received either 30 µmol/kg MIV-711 (synthesized by Medivir and given as a suspension in 1% Methocel A4C; n = 15) or vehicle (1% Methocel A4C; n = 15) by orogastric gavage once daily in the morning. The dose and dosing interval of MIV-711 was selected based on the potency of MIV-711 against dog cathepsin K enzyme (Ki=1.5 nmol/L, [28]) and the PK profile in normal beagle dogs. Dosing started the day before surgery and lasted through Day 28, the day before necropsy. Each dog was fasted for at least 12 h before dosing and food were returned approximately 4 h following test article administration.

Blood was collected on Days 1, 7, and 28 for evaluating the exposure of MIV-711. Blood was collected pre-dose and at 1, 2, 4, 8 and 24 h following oral administration. Blood samples were placed in tubes containing K_2_EDTA, and the samples were centrifuged within 30 min of blood collection at 2–8 °C for 10 min at approximately 3000 rpm. Plasma was separated and stored at − 70 °C until analysis.

Synovial fluid was collected from as many dogs as possible immediately before surgery and then again during necropsy from both operated and contralateral knees. The fluid was sampled directly without lavage using a syringe. The synovial fluid was transferred into tubes containing EDTA. The samples were spun in a centrifuge, and the supernatant was collected and stored at − 70 °C until analysis.

Urine samples were collected from the animals 1 to 3 days before surgery and treatment (baseline sample), 4 to 7 days after the start of treatment (Day 7 sample) and 26 to 28 days after treatment (Day 28 sample). Dogs were housed in metabolic cages overnight during urine collection. If no urine was collected from the metabolic cage, then the dog was manually restrained, and up to 15 mL of urine was collected by cystocentesis. Urine was centrifuged at 2–8 °C for 10 min at approximately 3000 rpm. Urine supernatant was collected and stored at − 70 °C until analysis.

#### Gross macroscopic scoring

All animals were humanely euthanized on Day 29 to collect specimens for pathology. Following sedation with acepromazine, euthanasia was performed via injection of a pentobarbital-based euthanasia solution (≥ 88 mg/kg pentobarbital). At study termination, the left (operated) knee was opened, disarticulated and the lesions on the medial tibia and femur measured, described and photographed. The observers were blinded to the treatment. Photographs were used (with or without India Ink lesion enhancement) to determine % area of lesions on each medial tibial plateau. Lesions were classified as more severe or less severe depending on the intensity of India ink staining or perception of depth and areas for each calculated. Gross cartilage lesions were measured (length × width) and described as superficial, moderate, or deep. Gross subjective cartilage degeneration scores were assigned for the medial and lateral tibial and femoral compartments as follows based on lesion measurements (see above): 0: normal, 1: superficial degeneration up to 10 mm^2^, 2: superficial degeneration greater than 10 mm^2^, 3: moderate depth degeneration up to 15 mm^2^, 4: moderate depth degeneration greater than 15 mm^2^. Subjective scores for the various regions were then used to generate an overall index of subjective cartilage degeneration for each region for each animal. Actual measurements of cartilage lesions were used to derive a calculated set of data points based on length × width × depth score (with depth score of 1 = superficial, 2 = medium, and 3 = deep, based on gross description). Percent of more or less severe tibial lesion area was determined from photographs taken at necropsy.

#### Histology

Details on histology can be found in Additional file 2.

### Assessment of biomarkers and MIV-711 exposure

In rabbits, conventional CTX-I assays to assess bone resorption cannot be used due to species differences. Instead, urinary helical peptide (HP-1) concentrations were measured in rabbits using the MicroVue Helical Peptide kit (Ref: 8022) from Quidel (San Diego, CA) according to the instructions supplied by the manufacturer. In dogs, urinary levels of C-terminal telopeptide of collagen type I (CTX-I) were measured using a commercially available kit (Urine BETA CrossLaps ELISA, Ref: AC-05F1, IDS Nordic Bioscience Diagnostics A/S). In rabbits and dogs, urinary concentrations of C-terminal telopeptide of collagen type II (CTX-II) were measured using the Urine CartiLaps EIA kit (Ref: AC-10F1) from Immunodiagnostics Systems (IDS, Herlev, Denmark) according to the instructions supplied by the manufacturer. In dogs, CTX-II levels were also measured in synovial fluid. Before ELISA analysis, 95 µL of synovial fluid was mixed with 5 µL of hyaluronidase (100 U/mL, Sigma, H-3884). The mixture was incubated overnight at 35 °C. The mixture was then used to measure CTX-II levels (Serum Pre-Clinical CartiLaps ELISA, Ref: AC-08F1, IDS Nordic Bioscience Diagnostics A/S). The creatinine levels in urine were determined at Swedish University of Agricultural Sciences (Uppsala, Sweden), using the enzymatic assay (Cat No. 8L24-01) on the Abbott system Architect c4000.

To measure MIV-711 concentrations, 10–50 µL of plasma or synovial fluid was mixed with 30–150 µL of acetonitrile, samples were centrifuged (10 min, 20,000*g*, 7 °C) and 5 µL of the supernatant was injected onto the LC–MS/MS system. The lower limit of quantification (LLOQ) was 1 nmol/L.

### Statistical analysis

Biomarker data was evaluated by two-way ANOVA, followed by Bonferroni’s or Dunnett’s correction for multiple comparisons when appropriate, using GraphPad Prism, version 6 (San Diego, CA). Synovial MIV-711 exposure data was evaluated by Student’s paired t-test. Data from µCT in rabbits was evaluated by one-way ANOVA followed by Dunnett’s correction for multiple comparisons (GraphPad). Macroscopic parameters in dogs were compared using Student’s unpaired t-test. The exposure data, biomarker data and macro- and microscopic data from the dog study were also imported into R (R Foundation for Statistical Computing Version 2.15.0, Vienna, Austria). A principal components analysis was performed using procedure prcomp. Derived scores were subjected to t-tests in Microsoft Excel 2007. Data are expressed as mean ± SEM, n = number of animals. A *p*-value < 0.05 was considered statistically significant.

## Results

### Effect of MIV-711 in the rabbit ACLT model

#### Pharmacokinetics of MIV-711 in rabbits

**Fig. 1:**
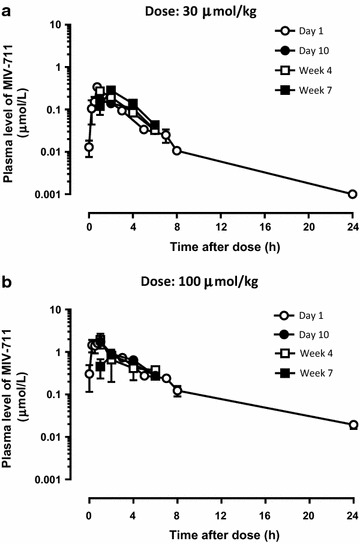
Plasma concentrations of MIV-711 in rabbits on Day 1, Day 10, Week 4 and Week 7 after oral administration of MIV-711 once daily at **a** 30 µmol/kg and **b** 100 µmol/kg. Mean ± SEM, n = 3–7 per time point

The plasma concentrations of MIV-711 were determined on four different occasions during the study. The plasma concentrations were similar after a single dose of MIV-711 and after repeated dosing for 7 weeks (Fig. 1). The C_max_ was 0.25 and 1.8 µmol/L after a single administration of MIV-711 at 30 and 100 µmol/kg, respectively. The corresponding area under the curve (AUC)_0–24 h_ was 0.78 and 7.0 µmol * h/L for the low and high dose, respectively.

#### Effect of MIV-711 on the bone resorption biomarker HP-1

**Fig. 2:**
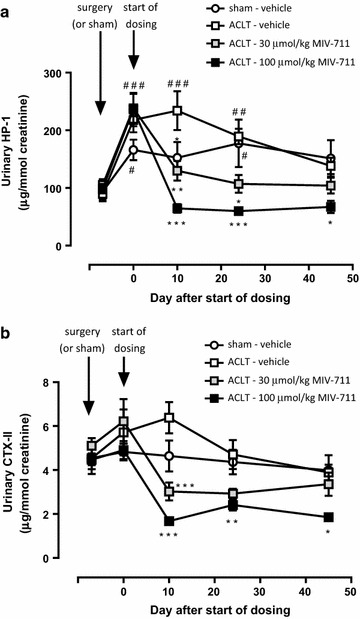
Effect of MIV-711 on urinary concentrations of **a** the bone resorption biomarker HP-1 and **b** the cartilage degradation biomarker CTX-II. Mean ± SEM, n = 5–7 per time point. *p < 0.05, **p < 0.01, ***p < 0.001 vs. ACLT-vehicle group. ^#^*p *< 0.05, ^##^*p *< 0.01, ^###^*p *< 0.001 vs. respective baseline

Urinary HP-1 levels were similar amongst the four groups at baseline (Fig. 2a). HP-1 levels in sham-operated vehicle-treated animals increased approximately 1.8-fold (*p* < 0.05) during the 7 days after surgery and stayed at 1.6- to 1.9-fold above baseline for the remainder of the study. Urinary HP-1 levels in rabbits subjected to ACLT increased app. 2.3- to 2.4-fold vs. baseline (*p* < 0.001) during the 7-day period after surgery. HP-1 levels in ACLT rabbits treated with vehicle increased more than in sham-operated rabbits treated with vehicle after 10 days of dosing (2.6-fold vs. 1.6-fold, *p* < 0.05). After 4 weeks of vehicle treatment and onwards, HP-1 levels were similar between sham and ACLT animals.

MIV-711 decreased HP-1 levels in a dose-dependent manner (Fig. 2a). After 10 days of dosing, HP-1 levels were reduced by 45% (*p* < 0.01) and 72% (*p* < 0.001); after 4 weeks by 43% (*p* < 0.05) and 68% (*p* < 0.001) and after 7 weeks by 25% (*p* > 0.05) and 51% (*p* < 0.05) in ACLT rabbits treated with MIV-711 at 30 and 100 µmol/kg, respectively, compared to ACLT rabbits treated with vehicle. HP-1 levels in the MIV-711-treated ACLT groups were also lower than HP-1 levels in sham-treated animals.

#### Effect of MIV-711 on the cartilage degradation biomarker CTX-II

Baseline urinary CTX-II levels were similar amongst the four groups at baseline (Fig. 2b) and did not seem to be affected by sham or ACLT surgery. Urinary CTX-II levels were similar in vehicle-treated sham and ACLT animals throughout the study. MIV-711 decreased CTX-II levels in a dose-dependent manner (Fig. 2b). After 10 days of dosing, CTX-II levels were reduced by 53% (*p* < 0.001) and 74% (*p* < 0.001); after 4 weeks, 38% (*p* > 0.05) and 49% (*p* < 0.01) and after 7 weeks, 14% (*p* > 0.05) and 52% (*p* < 0.05) in ACLT rabbits treated with MIV-711 at 30 and 100 µmol/kg, respectively, compared to ACLT rabbits treated with vehicle. CTX-II levels in the MIV-711-treated ACLT groups were also lower than CTX-II levels in sham-treated animals.

#### Effect of MIV-711 on subchondral bone structure as determined by µCT

**Fig. 3:**
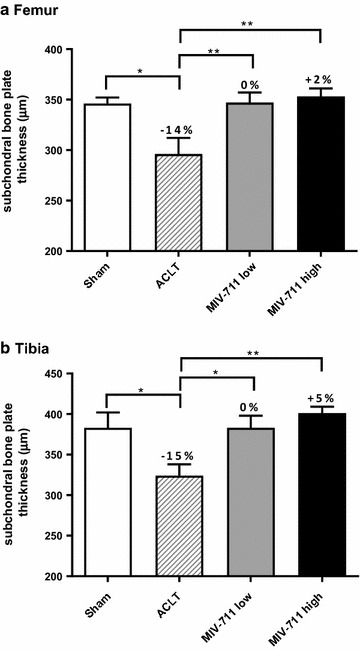
Effect of MIV-711 on **a** femur subchondral bone plate thickness and **b** tibia subchondral bone plate thickness. Note: Y-axis is hatched at 200 µm. Mean ± SEM, n = 6–7. *p < 0.05, **p < 0.01 vs. ACLT-vehicle group

ACLT surgery significantly reduced the total thickness of the subchondral bone plate in the femur (by 14% vs. sham, Fig. 3a). The total thickness of the bone plate was restored to sham levels in both groups receiving MIV-711 (0 and + 2% difference vs. sham for low and high dose MIV-711, respectively). Loss of bone was observed in both medial (− 12%), and lateral (− 18%) areas of the femur in response to ACLT and MIV-711 treatment restored bone plate thickness in both areas (Table 1). ACLT surgery also significantly reduced the total thickness of the subchondral bone plate in the tibia (by 15% vs. sham, Fig. 3b). The thickness of the bone plate was restored to sham levels in both groups receiving MIV-711 (0 and + 5% difference vs. sham for low and high dose, respectively). Unlike the femur, the bone was lost predominately in the lateral region (− 26% thickness decrease) of the tibia while only 6% of medial bone thickness was lost (Table 1). MIV-711 at both doses reversed the bone loss in both areas although the effect was only significantly different compared to vehicle for the lateral region of the tibia. As expected, the volume of the subchondral bone plate was reduced by ACLT and treatment with MIV-711 restored the volume, mainly in the lateral regions of femur and tibia (Table 1).

**Table 1:** Rabbit ACLT study—µCT data from subchondral bone plate

	Medial		Lateral		Total	
	Thickness (µm)	Volume (mm^3^)	Thickness (µm)	Volume (mm^3^)	Thickness (µm)	Volume (mm^3^)
Femur						
Sham-vehicle	368 ± 6	27.3 ± 1.0	325 ± 6*	23.6 ± 1.5	346 ± 6*	50.8 ± 2.5
ACLT-vehicle	324 ± 25	25.0 ± 2.1	268 ± 14	20.5 ± 1.2	296 ± 16	45.5 ± 3.1
ACLT-low dose	380 ± 13*	29.4 ± 0.8	316 ± 10	25.0 ± 0.6*	347 ± 10**	54.4 ± 0.8*
ACLT-high dose	388 ± 9*	29.9 ± 1.0	321 ± 11*	25.7 ± 1.2*	353 ± 8**	55.6 ± 2.0*
Tibia						
Sham-vehicle	450 ± 10	20.9 ± 0.6	330 ± 10*	19.4 ± 0.7*	383 ± 19*	40.3 ± 1.1*
ACLT-vehicle	424 ± 22	19.4 ± 1.2	243 ± 12	14.1 ± 0.9	324 ± 14	33.4 ± 1.7
ACLT-low dose	452 ± 15	20.6 ± 0.8	326 ± 18*	18.5 ± 1.3*	383 ± 15*	39.1 ± 2.0*
ACLT-high dose	462 ± 13	20.6 ± 0.5	353 ± 12*	20.7 ± 1.0*	401 ± 8**	41.2 ± 0.9*

* *p *< 0.05; ** *p *< 0.01 vs. ACLT-vehicle group

**Fig. 4:**
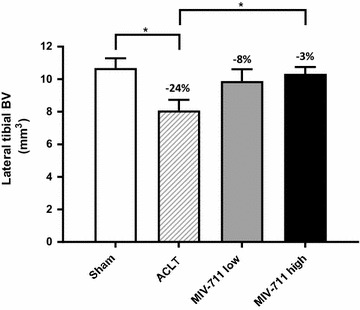
Effect of MIV-711 on tibial lateral bone volume. Mean ± SEM, n = 6–7. *p < 0.05 vs. ACLT-vehicle group

ACLT surgery also reduced trabecular bone volume in the lateral tibia (by 24% vs. sham, *p* < 0.05; Fig. 4 and Table 2) and this effect was significantly reversed by high dose MIV-711 (− 3% vs. sham, *p* < 0.05; Fig. 4). In other regions, the trabecular bone volume in ACLT control animals was lower than sham and MIV-711 attenuated bone volume loss in a dose-dependent manner in the medial tibia (vehicle: − 21% vs. MIV-711 high dose: − 2%), lateral femur (vehicle: − 19% vs. MIV-711 high dose: − 7%) and medial femur (vehicle: − 14% vs. MIV-711 high dose: − 7%) although these effects were not statistically significant (Table 2). As shown in Table 2 there were few statistically significant differences between groups. However, noteworthy trends that were consistent amongst the four regions include: (1) trabecular number and thickness were decreased, and trabecular separation was increased in the ACLT vehicle group compared to sham, (2) trabecular number and separation were both normalized by MIV-711 treatment at both doses, (3) tissue mineral density was decreased by approximately 5% in ACLT vehicle group compared to sham in all four regions. MIV-711 did not affect tissue mineral density in the femur but appeared to attenuate the decreased tissue mineral density in the tibia in a dose-dependent manner.

**Table 2:** Rabbit ACLT study—µCT data from trabecular bone

Parameter	Femur		Tibia	
	Medial	Lateral	Medial	Lateral
Trabecular bone volume (mm^3^)				
Sham-vehicle	7.8 ± 0.4	5.9 ± 0.3	9.7 ± 0.7	10.7 ± 0.6*
ACLT-vehicle	6.7 ± 0.3	4.8 ± 0.3	7.6 ± 0.7	8.1 ± 0.7
ACLT-low dose	7.0 ± 0.7	5.3 ± 0.5	8.9 ± 0.7	9.9 ± 0.7
ACLT-high dose	7.2 ± 0.5	5.5 ± 0.2	9.5 ± 0.5	10.3 ± 0.4*
Connective density (1/mm^3^)				
Sham-vehicle	8.00 ± 0.31	8.63 ± 0.50	5.79 ± 0.30*	11.35 ± 1.04*
ACLT-vehicle	9.30 ± 0.75	12.12 ± 1.65	9.93 ± 1.29	18.17 ± 2.35
ACLT-low dose	11.42 ± 1.61	13.51 ± 1.54	9.20 ± 1.08	16.55 ± 1.95
ACLT-high dose	9.14 ± 0.63	11.90 ± 1.51	8.47 ± 0.69	16.25 ± 2.01
Structure model index				
Sham-vehicle	0.35 ± 0.13	0.62 ± 0.09	0.53 ± 0.09	0.52 ± 0.11
ACLT-vehicle	0.30 ± 0.06	1.02 ± 0.09	0.79 ± 0.17	0.78 ± 0.19
ACLT-low dose	0.48 ± 0.07	0.73 ± 0.15	0.52 ± 0.16	0.51 ± 0.12
ACLT-high dose	0.40 ± 0.07	0.63 ± 0.11	0.41 ± 0.10	0.42 ± 0.11
Trabecular number (1/mm)				
Sham-vehicle	3.22 ± 0.28	2.32 ± 0.27	2.03 ± 0.15	2.88 ± 0.18
ACLT-vehicle	2.79 ± 0.10	2.19 ± 0.09	1.94 ± 0.17	2.63 ± 0.14
ACLT-low dose	3.02 ± 0.20	2.58 ± 0.16	2.05 ± 0.08	2.96 ± 0.15
ACLT-high dose	3.71 ± 0.68	2.55 ± 0.10	2.09 ± 0.15	3.01 ± 0.16
Trabecular thickness (mm)				
Sham-vehicle	0.24 ± 0.00	0.21 ± 0.01	0.28 ± 0.01	0.24 ± 0.01*
ACLT-vehicle	0.22 ± 0.01	0.19 ± 0.01	0.23 ± 0.01	0.19 ± 0.01
ACLT-low dose	0.22 ± 0.03	0.18 ± 0.01	0.25 ± 0.02	0.21 ± 0.01
ACLT-high dose	0.22 ± 0.02	0.19 ± 0.01	0.26 ± 0.00	0.22 ± 0.01
Trabecular separation (mm)				
Sham-vehicle	0.32 ± 0.03	0.48 ± 0.08	0.50 ± 0.04	0.35 ± 0.02
ACLT-vehicle	0.36 ± 0.01	0.46 ± 0.02	0.54 ± 0.05	0.39 ± 0.02
ACLT-low dose	0.34 ± 0.02	0.40 ± 0.03	0.49 ± 0.02	0.34 ± 0.02
ACLT-high dose	0.30 ± 0.03	0.40 ± 0.02	0.50 ± 0.04	0.34 ± 0.02
Bone surface (mm^2^)				
Sham-vehicle	103.6 ± 3.6	93.9 ± 4.8	116.8 ± 4.1	143.6 ± 4.6
ACLT-vehicle	101.7 ± 1.9	90.2 ± 2.3	119.2 ± 6.2	144.4 ± 4.4
ACLT-low dose	105.6 ± 3.8	101.2 ± 4.3	124.9 ± 3.3	153.6 ± 4.1
ACLT-high dose	105.2 ± 1.3	101.7 ± 2.9	124.9 ± 4.6	152.7 ± 4.1
Bone surface/volume (1/mm)				
Sham-vehicle	13.4 ± 0.2	16.0 ± 0.5	12.2 ± 0.6*	13.6 ± 0.6*
ACLT-vehicle	15.4 ± 0.8	19.3 ± 1.6	16.1 ± 1.1	18.5 ± 1.5
ACLT-low dose	16.0 ± 1.6	19.9 ± 1.3	14.6 ± 1.2	16.0 ± 1.2
ACLT-high dose	15.1 ± 1.0	18.7 ± 0.8	13.2 ± 0.2	14.9 ± 0.5
Tissue mineral density (mgHA/ccm)				
Sham-vehicle	733.2 ± 9.2	709.1 ± 8.9*	743.0 ± 7.8	717.8 ± 8.4
ACLT-vehicle	697.6 ± 8.6	670.5 ± 9.2	702.2 ± 11.4	680.2 ± 11.0
ACLT-low dose	698.4 ± 15.7	669.6 ± 8.4	714.1 ± 13.1	700.8 ± 10.6
ACLT-high dose	699.1 ± 10.3	673.6 ± 7.0	726.9 ± 5.5	707.0 ± 6.6

* *p *< 0.05; vs. ACLT-vehicle group

Osteophyte volumes in the femur and tibia were low in the sham surgery group and numerically higher in animals subjected to ACLT. However, due to the high variability of the osteophyte volumes in the ACLT groups, the comparisons were not statistically significant, and the volumes were not apparently affected by MIV-711 (*data not shown*).

#### Effect of MIV-711 on articular cartilage structure as determined by µCT

In femur, ACLT resulted in cartilage thickening, particularly on the posterior medial condyle, while in some regions cartilage was focally thinned or completely eroded, particularly on the abaxial aspect of the medial condyle. As shown in Table 3, ACLT increased total femur cartilage thickness (by 24%, *p* < 0.01) and cartilage volume (by 33%, *p* < 0.05) compared to sham controls. A significant increase was seen in both medial and lateral aspects of the femur. MIV-711 attenuated the ACLT-evoked increase in cartilage swelling in both aspects, especially laterally, however, the effects were not statistically significant and not obviously dose-dependent. The effects of ACLT were less prominent on tibia cartilage (11% increase in thickness and 13% increase in volume vs. sham, *p* > 0.05). The ACLT-evoked increase in tibial cartilage thickness was numerically attenuated by MIV-711 and reached statistical significance in the low dose group (*p* < 0.05, Table 3).

**Table 3:** Rabbit ACLT study—cartilage µCT data

	Medial		Lateral		Total	
	Thickness (µm)	Volume (mm^3^)	Thickness (µm)	Volume (mm^3^)	Thickness (µm)	Volume (mm^3^)
Femur						
Sham-vehicle	276 ± 5*	23.2 ± 0.9*	223 ± 8*	18.2 ± 1.5*	250 ± 3**	41.4 ± 2.0*
ACLT-vehicle	345 ± 15	31.1 ± 2.2	272 ± 15	23.9 ± 2.2	309 ± 14	55.0 ± 4.2
ACLT-low dose	310 ± 18	27.5 ± 2.6	257 ± 13	23.1 ± 1.1	283 ± 15	50.6 ± 3.5
ACLT-high dose	306 ± 12	27.2 ± 1.6	251 ± 12	23.0 ± 1.1	278 ± 11	50.2 ± 2.2
Tibia						
Sham-vehicle	558 ± 15	26.9 ± 1.0	349 ± 7	20.0 ± 0.6	444 ± 10	46.8 ± 1.4
ACLT-vehicle	611 ± 17	28.8 ± 1.2	402 ± 12	23.9 ± 1.2	494 ± 12	52.8 ± 2.2
ACLT-low dose	533 ± 26*	24.9 ± 1.5	349 ± 18	19.6 ± 1.3	432 ± 20*	44.5 ± 2.6
ACLT-high dose	539 ± 25	25.3 ± 1.2	369 ± 24	21.6 ± 2.0	445 ± 22	46.9 ± 3.1

* *p *< 0.05; vs. ACLT-vehicle group

** *P*<0.01 vs. ACLT-vehicle group

#### Effect of MIV-711 on subchondral bone and articular cartilage as determined by histomorphometry

The results of the histomorphometric analysis of bone and cartilage are summarized in Additional file 3: Table S2. Overall, the effects of MIV-711 at the microscopic level were not statistically significant. This could in part be explained by larger variations observed at the microscopic level, which may be due to where the section was taken. The ACLT-evoked bone loss in the subchondral bone plate and trabecular bone was prevented by MIV-711. Also, increased cartilage width was detected in most sections which appeared to be attenuated by MIV-711. MIV-711 did not affect osteophyte parameters or Mankin scores at the microscopic level (Additional file 3: Table S2).

### Effect of MIV-711 in the partial medial meniscectomy model in dog

#### Pharmacokinetics of MIV-711 in dogs

**Fig. 5:**
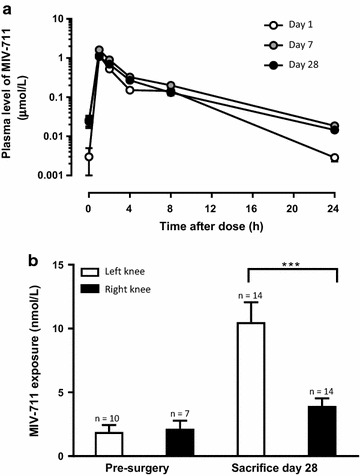
**a** Plasma concentrations of MIV-711 in dogs on Day 1, Day 7 and Day 28 and **b** synovial concentrations of MIV-711 at pre-surgery (24 h after the first dose of MIV-711) and on Day 29 (24 h after last dose of MIV-711). Mean ± SEM. Numbers above columns indicate number of animals sampled. ***p<0.001 vs. right knee

Plasma levels of MIV-711 in dogs after oral dosing at 30 µmol/kg were measured on Days 1, 7 and 28 after the start of dosing (Fig. 5a). Exposure of MIV-711 was consistent throughout the study. The mean C_max_ ranged between 1.1 and 1.6 µmol/L, and the mean AUC_0–24 h_ ranged between 3.6 and 6.1 µmol * h/L on the different PK sampling days. The exposure of MIV-711 in the synovial fluid of the injured (left) and non-injured (right) knee is shown in Fig. 5b. Synovial fluid was collected pre-surgery when possible (24 h after the first dose) and at necropsy (24 h after the last dose). MIV-711 levels were low but measurable. Interestingly, MIV-711 levels were fivefold higher (*p* < 0.001) in the injured left knee compared to the right knee at sacrifice.

#### Effect of MIV-711 on biomarkers

**Fig. 6:**
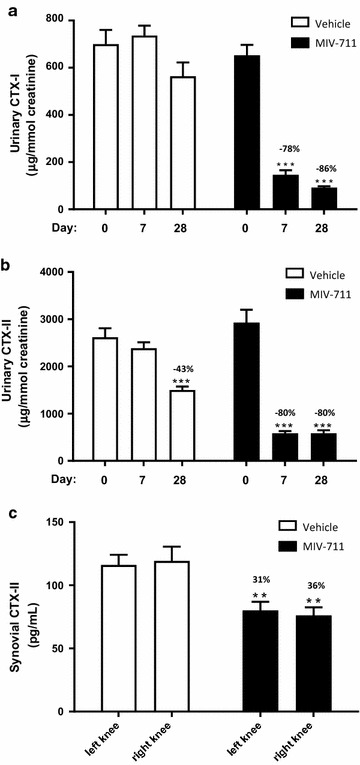
Effect of vehicle or MIV-711 (30 µmol/kg) on **a** urinary levels of CTX-I, **b** urinary levels of CTX-II and **c** synovial levels of CTX-II at necropsy (Day 28). Mean ± SEM, n = 15, **p < 0.01, ***p < 0.001 vs. baseline in **a**, **b** and **p < 0.01 vs. vehicle in **c**

Urinary CTX-I levels were similar in both groups at baseline (Fig. 6a). While vehicle-treated animals had similar urinary CTX-I levels throughout the study, MIV-711 treatment reduced the CTX-I levels by 78% (*p* < 0.001) and 86% (*p* < 0.001) on Days 7 and 28, respectively. Urinary CTX-II levels were similar between the groups at baseline and on Day 7 (Fig. 6b), while being 43% lower (*p* < 0.001) than baseline on Day 28 in vehicle-treated dogs. MIV-711 treatment reduced CTX-II levels by 80% (*p* < 0.001) on both Day 7 and Day 28. Synovial fluid was collected from the dogs in conjunction with surgery where possible. CTX-II levels were similar between the groups at baseline (data not shown), while at necropsy, synovial CTX-II levels were significantly reduced in both knees from MIV-711-treated animals (by 55–57% compared to baseline, *p* < 0.001). Synovial CTX-II levels were also reduced in vehicle-treated animals compared to baseline. However, CTX-II levels were 31–36% (*p* < 0.01) lower in MIV-711-treated animals compared to vehicle-treated at necropsy (Fig. 6c).

#### Effect of MIV-711 on macroscopic and microscopic scores

**Fig. 7:**
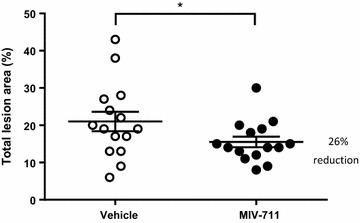
Effect of vehicle or MIV-711 (30 µmol/kg) on cartilage lesion area of the left tibia plateau. *p < 0.05

The macroscopic lesion scores in femur condyles and tibial plateaus are summarized in Table 4. MIV-711-treated animals had 25–37% lower macroscopic scores in the femur condyles and 13–33% lower macroscopic scores in the tibial plateaus. Due to the vast variations between animals, these changes were not statistically significant. The cartilage lesions in the tibia were also measured using imaging analysis. The proportion of cartilage affected by lesions in vehicle-treated controls was 21.0% ± 2.6, while the lesioned area in MIV-711 treated animals was 15.5% ± 1.4 (*p* < 0.05, Fig. 7). Sub-analysis demonstrated that severe tibial cartilage lesions were present in 6/15 dogs in the vehicle-treated group while being detected in 3/15 dogs in the MIV-711-treated group. The area of these severe lesions was 10 ± 4% in the six dogs from the vehicle group while being 2 ± 1% in the three dogs from the MIV-711 group (i.e. 80% lower, *p* = 0.08). The results of the microscopic scoring of cartilage and bone parameters are summarized in Additional file 4: Table S3. MIV-711 treated animals had a statistically significant decrease in cartilage degeneration scores in level 1 (anterior) of the tibia (by 41%), although overall means and other specific regions were not significantly affected (see Table S3a in Additional file 4). Cartilage degeneration scores were – 7 to 9% lower in MIV-711-treated animals compared to vehicle-treated animals without reaching statistical significance (Additional file 4: Table S3a). Similarly, no significant effects of MIV-711 on subchondral bone sclerosis and bone area were observed at the microscopic level (see Tables S3a and S3f in Additional file 4).

**Table 4:** Dog partial medial meniscectomy study—macroscopic scoring

Region	Scoring	Vehicle	MIV-711	%difference
Femur condyle	Subjective	1.80 ± 0.34	1.13 ± 0.32	− 37
Femur condyle	Calculated	12.13 ± 3.91	9.07 ± 4.78	− 25
Tibia plateau	Subjective	2.67 ± 0.32	2.33 ± 0.21	− 13
Tibia plateau	Calculated	35.69 ± 6.66	23.88 ± 3.17	− 33

#### Principal components analysis

Principal component analysis divided the variance into principal components. Score 1 accounted for 26% of the total variance, while score 2 accounted for 15%. Score 1 included gross scores, gross lesion area data, and some histological variables. Score 1 was significantly different between the vehicle and MIV-711-treated groups (*p* < 0.05). Score 2 was mainly loaded with biomarker data and was also significantly different (*p* < 0.001) between the vehicle and MIV-711-treated groups.

#### Comparison of exposure across species

**Fig. 8:**
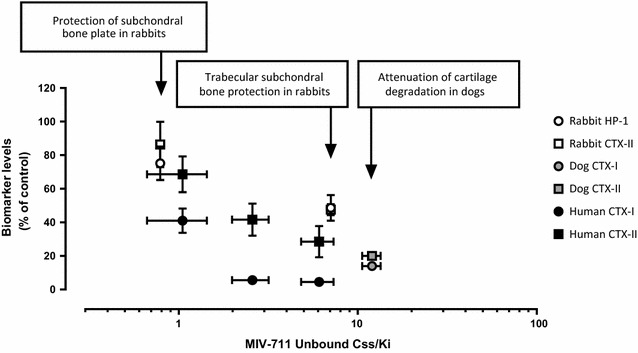
Unbound exposure corrected for cathepsin K potency vs. effect on biomarkers of bone resorption and cartilage degradation in various species. Circles represent bone resorption biomarkers (CTX-I and HP-1), while squares depict biomarkers of cartilage degradation (CTX-II). Rabbit biomarker data (open symbols) are from Week 7 and reflect % of vehicle control. Dog biomarker data (grey symbols) are from Day 28 and reflect % of baseline. Human biomarker data (black symbols) are from Day 7 and reflect % of baseline after 7-day dosing with 50, 100 and 200 mg MIV-711, respectively [30]. Potency corrected exposure data was calculated by taking the AUC_0–24 h_ of MIV-711 and dividing by 24 h to reach C_ss_ (steady state concentration). Unbound C_ss_ was obtained by correcting for protein binding for the various species. C_ss_ was then divided by the K_i_ of MIV-711 for the various species

The Ki values for MIV-711 against cathepsin K from human, dog, and rabbit are 0.98, 1.5 and 3.3 nmol/L, respectively [28]. The fraction of MIV-711 bound to plasma proteins in human, dog, and rabbit are 94, 90 and 92%, respectively. Figure 8 summarizes the potency-corrected exposures of unbound MIV-711 required for effects on biomarkers and structural outcomes in the preclinical models described in this article and are related to the exposure and effect on biomarkers observed in human in initial clinical studies with MIV-711 [30]. The data at hand suggest that relatively low exposures of MIV-711 are sufficient for protective effects on bone, while higher concentrations and larger reductions in biomarker levels may be required for attenuation of cartilage degeneration in the dog. The graph also shows that these concentrations of MIV-711 in preclinical models are clinically relevant and associated with similar reductions in biomarkers in healthy volunteers.

## Discussion

The current article demonstrates that the potent and selective cathepsin K inhibitor MIV-711 reduces biomarkers of bone resorption and cartilage degradation in the ACLT model in rabbit and the partial medial meniscectomy model in dog. The doses of MIV-711 used resulted in near complete protection of subchondral bone in rabbits. MIV-711 also attenuated cartilage lesions in the dog model and decreased cartilage swelling in the rabbit model. The potency-corrected exposures of MIV-711 reached in these two preclinical models of OA were in the same range as exposures reached in human and that have been shown to be safe and well tolerated. The reduction of biomarkers in dog and rabbit were of similar magnitude as in human, thus offering a translational potential for MIV-711.

The ACLT model in the rabbit is characterized by subchondral bone loss together with cartilage swelling and focal degradation [31–33]. In the current study, ACLT surgery significantly elevated HP-1 levels, which may be indicative of an early increase in bone resorption, either due to the induced joint instability or altered loading patterns on the knee joint after surgery [33]. MIV-711 reduced both HP-1 and CTX-II biomarkers in a parallel and dose-dependent manner, demonstrating successful target engagement of cathepsin K at the doses used in the rabbit. MIV-711 exposure was consistent throughout the 7-week dosing period. The effect of MIV-711 on the biomarker levels, measured as % of the vehicle, decreased numerically over time, but this was more related to decreases in biomarker levels in the vehicle group rather than loss of target engagement.

Analysis using µCT showed that the therapeutic administration of MIV-711 reversed the effects of ACLT on most subchondral bone parameters in the rabbit, with administration starting 7 days after surgery, at a time point where the HP-1 levels were close to maximal. Interestingly, in most cases, the effects of MIV-711 on the subchondral bone plate were not dose-dependent, with complete reversal of bone loss observed already at the low dose. Similar effects on the subchondral bone plate were seen with the low and high doses of MIV-711, even though the high dose resulted in tenfold higher exposures and twofold larger reductions in the HP-1 levels. In contrast, it appeared that there were some dose-dependent effects on trabecular bone, with the higher dose of MIV-711 producing more consistent protective effects. This suggests that relatively low doses of MIV-711 are sufficient for protective effects on the subchondral bone plate while higher doses are required for protection of underlying trabecular bone. In dog OA models it has been suggested that bone loss in the subchondral bone plate correlates well with cartilage changes and is thus more intrinsically linked to the OA process than the trabecular bone loss which may reflect less loading of the destabilized knee joint [34, 35].

Although areas of focally eroded cartilage were present in response to ACLT in rabbits, the current study shows that ACLT also evoked increases in cartilage thickness and volume, in all four areas examined, when quantified by µCT. Our results are in line with recent data demonstrating increases in the femur and tibia cartilage thickness at 8 weeks following ACLT in rabbits using µCT [36]. Similar changes in cartilage volume have been demonstrated in partial medial meniscectomy [32] and ACLT [33] rabbit models of OA using MRI. The increase in cartilage volume has been attributed to cartilage swelling and has been suggested to precede the loss of subchondral bone [32]. Interestingly, cartilage swelling was numerically reduced by both doses of MIV-711 in all areas examined although the effects only reached statistical significance in the medial cartilage of the tibia plateau with the low dose. Since there was a lack of dose-dependency and cartilage swelling was not completely reversed, it may suggest that the reduced cartilage swelling is indirectly due to improved quality of the underlying subchondral bone plate. If cartilage swelling precedes subchondral bone loss as in the study by Calvo et al. [32] and if the subchondral bone is the main target for MIV-711, then it is not surprising that the subchondral bone plate was wholly protected while cartilage swelling was merely attenuated. Unlike µCT which quantifies all articular cartilage, histological assessment depends on the specific section analyzed. Since the cartilage parameters analyzed using Mankin scores were very focal, we observed large degrees of variation precluding any detection of efficacy with MIV-711. An increased osteophyte volume in ACLT rabbits was quantified using µCT and histomorphometry but was not affected by MIV-711 treatment. The effects of anti-resorptive drugs on osteophyte formation in experimental OA models have been variable (see [37] for a review). For instance, in the dog ACLT model, the bisphosphonate NE-10035 was shown to inhibit trabecular bone resorption but not affect osteophyte formation when treatment was started the day after surgery [38], while alendronate inhibited osteophyte formation in the rat ACLT model when treatment was started before surgery [39]. This could be due to that osteophyte formation can be detected quickly after ACLT (within days) and that earlier interventions or longer treatment times may be required to observe an effect [37]. However, Hayami et al. [27] showed that cathepsin K inhibition could reduce osteophyte formation using a similar treatment protocol as in the current study.

Unlike the ACLT model in rabbits and dogs, the partial medial meniscectomy model has limited effects on subchondral bone and is more reflective of a model of cartilage degeneration [32, 40]. In the current dog study, neither urinary CTX-I or CTX-II levels increased in response to the surgery, similar to previous findings [26]. In contrast, urinary CTX-II levels were decreased in vehicle-treated animals after 28 days. This may be due to the age of the animals (6–8 months) in this study. Although growth plates were closed at this age, there could still be ongoing growth plate activity giving rise to relatively high CTX-II levels which would subsequently decrease with age and during the study [41]. MIV-711 reduced the CTX-I and CTX-II biomarkers measured in urine by 80–86% at the sacrifice which is consistent with the effects observed by the cathepsin K inhibitor SB553484 [26]. Concentrations of MIV-711 were also detected in synovial fluid, and CTX-II levels in this matrix were also reduced in response to MIV-711 treatment. The concentrations of MIV-711 in synovial fluid were low, but this is to be expected since the exposure was measured at trough, i.e. 24 h after the dose. Interestingly, significantly higher MIV-711 levels were detected in the injured joint compared to the contralateral control joint. This might be due to increased vascularization of the injured joint enabling more MIV-711 to reach this site, or to a higher degree of partitioning of non-basic MIV-711 into the joint where a pH as low as 5.5 has been measured in OA cartilage surface [17]. However, the relevance of increased MIV-711 exposure in the injured joint is difficult to appreciate concerning efficacy since the CTX-II reduction was similar in both joints.

Macroscopic and microscopic lesion scores in femur condyles and tibial plateaus in the dog were consistently reduced in response to MIV-711 treatment although the reductions were of borderline statistical significance when compared individually. Since many of the 101 variables analyzed were correlated, principal components analysis was used to derive scores that are combinations of the correlated individual variables. The first two scores explained 26 and 15% respectively of the total variance in the dataset, and both were significantly different between treatment groups. The first score was derived primarily from gross and histological morphological variables, and the second from PK and biomarker data, indicating that in this analysis of the aggregated data, treatment had a significant effect on both morphologic and biomarker endpoints. The magnitude of reduction at the macroscopic level was similar to the data observed with the cathepsin K inhibitor SB553484 [26]. However, although SB553484 is almost 30-fold more potent than MIV-711 against canine cathepsin K (0.055 nmol/L vs. 1.5 nmol/L), the daily doses of SB553484 were sixfold higher, and plasma exposures were not reported. Also, SB553484 has only marginal selectivity towards cathepsin K versus other cathepsins (15-fold, vs. > 1300-fold for MIV-711) making the contribution of cathepsin K in these two studies difficult to compare. In the study by Settle et al. [42] the protective effects on cartilage were more significant when using the MMP-13 inhibitor PF152. To our knowledge neither SB553484 nor PF152 has progressed into clinical development, thus making a translation of the preclinical data to the clinical relevance of the doses used and the effects achieved in the preclinical models difficult. In the current study, however, the potency-corrected exposures of MIV-711 in the dog are clinically relevant, since they are known to be safe, have been shown to engage cathepsin K in healthy volunteers and post-menopausal women and are intended to be reached in ongoing human studies in OA patients. Since the dog partial medial meniscectomy model has limited bone loss [32, 40], the attenuated cartilage degradation with MIV-711 may suggest direct effects of cathepsin K on cartilage per se. Indeed, cathepsin K can cleave collagen type II in vitro [18, 43]. However, it cannot be excluded that both an indirect effect on bone and a direct effect on cartilage could be involved.

Some anti-resorptive drugs have demonstrated encouraging signs of efficacy in clinical trials in OA and have also been effective in preclinical models of OA. The preclinical effects on bone and cartilage by these agents are in line with our data. Low doses of alendronate prevented bone loss in the estrogen-deficient ovariectomized (OVX) model in the rat, but did not prevent cartilage degradation in ACLT rats [39], and instead higher doses of alendronate were required to demonstrate protection of cartilage. Studies with the cathepsin K inhibitor L-006235 reveal a similar pattern, with complete protection of bone seen in OVX rabbits and ACLT rabbits using a dose of 10 mg/kg [27, 44], while higher doses of 50 mg/kg were needed to show some degree of cartilage protection [27]. In the current article, protective effects on the subchondral bone plate in the rabbit were achieved at relatively low plasma exposures and relatively low effects on biomarkers as summarized in Fig. 8. These exposures seemed to also offer attenuated cartilage swelling in the rabbit model. Higher exposures and more prominent effects on biomarkers seemed to be required for trabecular bone protection and consistent cartilage protective effects as seen in the dog model. Thus, it is essential to consider not only the agent and its mechanism of action but also the dose used, the exposure reached, and the target engagement achieved. Taken together, our results together with others suggest that with anti-resorptives, like bisphosphonates and cathepsin K inhibitors, relatively low doses offer bone protection while higher doses may be required for cartilage protection. If this reasoning is correct, then it is important to evaluate bone-acting agents like cathepsin K inhibitors at doses that are sufficiently high to have a meaningful effect on biomarkers of cartilage degradation, and that doses that are effective for the treatment of osteoporosis may be too low to achieve optimal effects in OA patients.

The effects of MIV-711 on biomarkers preclinically and clinically provide considerable translational value. The bone resorption biomarker HP-1 has not been used much in clinical studies, but rather as an alternative bone resorption biomarker in rabbits since conventional CTX-I assays cannot be used due to species differences. Nonetheless, urinary HP-I levels have been shown to be highly correlated to urinary CTX-I levels and treatment with alendronate reduced CTX-I and HP-I levels in man to a similar degree [45]. In the study by Hayami et al. [27], a dose of L-006235 providing a 60% reduction of HP-1 was sufficient for the protection of bone loss as assessed by histomorphometry. Our data suggest that even lower reductions of HP-1 translate into structural improvements on subchondral bone in rabbits. Figure 8 shows that these exposures can be reached in human and at reasonable doses.

The rapid degenerative process induced in the surgical models used in the current study is unlike the slow progression of disease that characterizes OA, and these rapid changes may, therefore, limit the efficacy of anti-resorptive compounds like MIV-711. However, it is difficult to speculate as to what degree of joint protection is required for translation into a clinical effect. Higher degrees of bone and cartilage protection could perhaps have been reached by further increasing the dose of MIV-711. However, the fact that the decreases in biomarkers were near maximal suggests that there is likely to be a limited additional benefit on the joint structure by further increasing the doses of MIV-711. Lack of clinically relevant exposures is often a shortcoming in preclinical studies trying to provide translational relevance to humans [46]. As shown in Fig. 8, MIV-711 has the potential to reach exposures in man that demonstrated structural protection in these preclinical models, and its effect on biomarkers enables the doses to be selected from early human studies to be tested in the longer-term clinical studies that will be required to show a disease-modifying effect in OA patients. In fact, the recent results from the initial Phase IIa study with MIV-711 in knee OA patients demonstrated significant effects on bone and cartilage structural endpoints (as assessed by MRI) after 6 months and the beneficial effects on the joint structure were associated with substantial reductions in the CTX-I and CTX-II biomarker levels [29]. The results suggest that the structural protection observed by MIV-711 in the preclinical OA models indeed translates into disease-modifying effects in OA patients.

## Conclusions

The potent and selective cathepsin K inhibitor MIV-711 attenuates joint pathology and reduces biomarkers of bone resorption and cartilage degradation in experimental models of OA. Similar exposures and changes in biomarker levels can be achieved in man and thus supports the potential of MIV-711 as a disease-modifying agent for the treatment of OA.

## Additional files

**Additional file 1.** Rabbit anterior cruciate ligament transection model—histology.

**Additional file 2.** Dog partial medial meniscectomy model—histology.

**Additional file 3: Table S2.** Rabbit anterior cruciate ligament transection model - histomorphometry data summary.

**Additional file 4: Table S3.** Dog partial medial meniscectomy model - summary of microscopic pathology.

## Abbreviations

ACL: anterior cruciate ligament
ACLT: anterior cruciate ligament transection
ANOVA: analysis of variance
AUC: area under the curve
C_max_: maximum concentration
CTX-I: C-terminal telopeptide I
CTX-II: C-terminal telopeptide II
EDTA: ethylenediaminetetraacetic acid
EIA: enzyme immune assay
ELISA: enzyme-linked immunosorbent assay
HP-1: helical peptide
IACUC: Institutional Animal Care and Use Committee
Ki: inhibitory constant
LC–MS/MS: liquid chromatography dual mass spectrometry
LLOQ: lower limit of quantitation
µCT: microcomputed tomography
MRI: magnetic resonance imaging
OA: osteoarthritis
OVX: ovariectomized
PK: pharmacokinetic
ROI: region of interest
WOMAC: Western Ontario and McMaster Universities Osteoarthritis Index
