# Supplementary material for: The selective cathepsin K inhibitor MIV-711 attenuates joint pathology in experimental animal models of osteoarthritis
Source: J Transl Med. 2018 Mar 9;16:56. doi: 10.1186/s12967-018-1425-7 (PMC5845353; doi:10.1186/s12967-018-1425-7)
Supplement: Supplementary file 1 — Additional file 1. Rabbit anterior cruciate ligament transection model—histology. [file 12967_2018_1425_MOESM1_ESM.docx]

**Additional file 1**

**Rabbit anterior cruciate ligament transection model – Histology**

The femora and tibias were decalcified, embedded in paraffin, sectioned at 5-7 µm at two different levels separated by approximately 2 mm, and stained with safranin O and toluidine blue.

**Histological Evaluation**

Both tibial plateaus and femoral condyles were subjected to semi-quantitative scoring using a modified Mankin scoring system. The scoring scheme is shown in Table 1. Slides were evaluated in animal ID order, i.e. in random order with respect to treatment, so that the reader was effectively blinded to treatment assignment.

**Histomorphometric Evaluation**

Both tibial plateaus and femoral condyles (i.e. four joint compartments) were also subjected to histomorphometric analysis of cartilage, subchondral bone, cancellous bone, and osteophytes. The measurements were made using OsteoMeasureXP version 3.3.0.2 as follows:

- Uncalcified cartilage area was measured by tracing the articular surface (cartilage perimeter) and bone or calcified cartilage/uncalcified cartilage interface (cartilage boundary).
- Cartilage width was measured by point to point measurement at intersections between a sampling grid with approximately 0.5 mm spacing and the cartilage boundary. The intercepts measured were orthogonal to the cartilage boundary and ended at the cartilage perimeter. At intersections where the cartilage width was zero due to erosion, a value of zero was recorded.
- Subchondral bone width was measured by point to point measurement at intersections between a sampling grid with approximately 0.5 mm spacing and the cartilage boundary. The intercepts measured were orthogonal to the cartilage boundary and ended at the first marrow cavity encountered that had at least one dimension approximately equivalent to nearby trabecular thickness.
- Osteophyte area was measured for abaxial osteophytes. On the femur, osteophytes associated with the condyles but not with the trochlear grooves were measured.
- Bone area (including osteoid) and perimeter were measured in a 3.8 X 1.5 mm ROI centered approximately 1 mm below the bone/cartilage interface. This ROI was intended to be equivalent to the subchondral cancellous bone VOI measured by microCT. Bone volume (BV/TV), trabecular thickness (Tb.Th), trabecular number (Tb.N) and trabecular separation (Tb.Sp) were derived from these measurements using standard formulae.

Slides were evaluated in animal ID order, i.e. in random order with respect to treatment, so that the measurer was effectively blinded to treatment assignment.

# Table S1. Modified Mankin Scoring Scheme

| Parameter Group | Parameter | Scoring | Morphological finding |
| --- | --- | --- | --- |
| Cartilage | Safranin O Staining | 0 | Uniform staining throughout articular cartilage |
|  |  | 1 | Loss of staining in the upper 1/3 of hyaline cartilage, <50% the length of condyle or plateau |
|  |  | 2 | Loss of staining in the upper 1/3 of hyaline cartilage, ≥50% the length of condyle or plateau |
|  |  | 3 | Loss of staining in the upper 2/3 of hyaline cartilage, <50% the length of condyle or plateau |
|  |  | 4 | Loss of staining in the upper 2/3 of hyaline cartilage, ≥50% the length of condyle or plateau |
|  |  | 5 | Loss of staining in all of the hyaline cartilage, <50% the length of condyle or plateau |
|  |  | 6 | Loss of staining in all of the hyaline cartilage, ≥50% the length of condyle or plateau |
|  | Structure | 0 | Normal |
|  |  | 1 | Surface irregularities |
|  |  | 2 | Fissures in <50% of surface^1^ |
|  |  | 3 | Fissures in ≥50% of surface^1^ |
|  |  | 4 | Erosion of 1/3 hyaline cartilage over <50% of the surface |
|  |  | 5 | Erosion of 1/3 hyaline cartilage over ≥50% of the surface |
|  |  | 6 | Erosion of 2/3 hyaline cartilage over <50% of the surface |
|  |  | 7 | Erosion of 2/3 hyaline cartilage over ≥50% of the surface |
|  |  | 8 | Full depth erosion of hyaline cartilage over <50% of the surface |
|  |  | 9 | Full depth erosion of hyaline cartilage over ≥50% of the surface |
|  |  | 10 | Full depth erosion of hyaline and calcified cartilage to the subchondral bone over <50% of surface |
|  |  | 11 | Full depth erosion of hyaline and calcified cartilage to the subchondral bone over ≥50% of surface |
|  | Chondrocyte density^2^ | 0 | No decrease in cells |
|  |  | 1 | Focal decrease in cells |
|  |  | 2 | Multifocal decrease in cells |
|  |  | 3 | Multifocal confluent decrease in cells |
|  |  | 4 | Diffuse decrease in cells |
|  | Cluster formation | 0 | Normal |
|  |  | 1 | <4 clusters |
|  |  | 2 | ≥4 but <8 clusters |
|  |  | 3 | ≥8 clusters |
| Bone | Osteophytes | 0 | Absent |
|  |  | 1 | Mild |
|  |  | 2 | Moderate |
|  |  | 3 | Severe |

# ^1^Artifact definition: fissure but no hypercellularity or loss of safranin O adjacent to fissure.

# ^2^Focal: observed at one site on section; multifocal: observed at >1 site; multifocal confluent: observed at multiple sites that are in contact.

**Statistical analyses**

All statistical analyses were performed using R, version 3.0 or higher (R Foundation for Statistical Computing, Vienna, Austria, <http://www.R-project.org/>). Mean, median, standard deviation, standard error of the mean and group size were calculated for all quantitative data.

For each continuous variable, normality was tested for each treatment group using the Shapiro-Wilk test and homogeneity of variance was tested using Levene’s test. For data that passed both tests, a one-way ANOVA was performed followed by Dunnett’s test to compare each treatment group to a single control group (Group 2; ACLT + vehicle) if the overall treatment effect was significant by ANOVA.

For non-continuous variables such as Mankin scores, and data that fail one or more normality or homogeneity of variance test, non-parametric tests were used. Data were analyzed using the Kruskal-Wallis test. If the overall treatment effect was significant, comparisons of interest were tested according to the procedure of Siegel and Catellan (1988, Non parametric statistics for the behavioulra sciences. MacGraw Hill Int., New York. pp 213-214).

All tests were considered statistically significant at the 5% probability level.
