# Supplementary material for: The selective cathepsin K inhibitor MIV-711 attenuates joint pathology in experimental animal models of osteoarthritis
Source: J Transl Med. 2018 Mar 9;16:56. doi: 10.1186/s12967-018-1425-7 (PMC5845353; doi:10.1186/s12967-018-1425-7)
Supplement: Supplementary file 2 — Additional file 2. Dog partial medial meniscectomy model—histology. [file 12967_2018_1425_MOESM2_ESM.docx]

**Additional file 2**

**Dog partial medial meniscectomy model - Histology**

**Processing of Left Knee Joint**

The proximal end of the left tibia and the distal end of the left femur were removed using a hacksaw and collected into 10% neutral buffered formalin. Bones were allowed to fix for 5 days and then the tissues were placed in formic acid decalcifier (5-20%). After decalcification, the surface area at risk for lesion development of the tibia (medial and lateral plateaus) and femur (medial and lateral condyles) was divided into 3 slabs (anterior to posterior) approximately 2-4 mm thick for processing, embedding, sectioning (8 µm) and staining (Toluidine Blue). The lesions are in reasonably consistent locations in all animals, therefore, the same surfaces and area were consistently embedded for all animals. Blocks containing tissue were numbered 1-3 with 1 being the most anterior piece. In all cases, the anterior aspect of the tissue was embedded down in the cassette for sectioning.

**Cartilage degeneration and osteophytes**

Compartmentalized lesions on medial tibia and medial femur (cartilage degeneration and osteophytes) were scored for each of the 3 (anterior to posterior) sections using the following system:

- 0=no degeneration
- 1=minimal degeneration, chondrocyte and proteoglycan loss with or without fibrillation involving the superficial zone or extending into the upper 10% of the cartilage thickness or at least 5% but not more than 10% overall total proteoglycan and cell loss in the zone if lesion is focal and deeper in some areas. Deeper and more extensive proteoglycan loss only without any evidence of chondrocyte loss and collagen damage is also given a score of 1 as this is a minor and reversible change
- 2=mild degeneration, chondrocyte and proteoglycan loss with fibrillation involving mainly the upper 11-25% of cartilage thickness, fibrillation generally superficial (upper 10%) while chondrocyte and proteoglycan loss extend into approximately 11-25% of the cartilage depth, or 11-25% overall chondrocyte and proteoglycan loss in the zone if lesion is focal and deeper in some areas. Deeper areas of proteoglycan loss only, without chondrocyte loss or collagen disruption (reversible changes) may be present
- 3=moderate degeneration, chondrocyte and proteoglycan loss with fibrillation extending well into the midzone and generally affecting 1/2 (50%) of the total cartilage thickness, fibrillation/collagen damage generally extends into upper 25% while chondrocyte and proteoglycan loss extend through 50% thickness, or 26-50% overall chondrocyte and proteoglycan loss in the zone if lesion is focal and deeper in some areas. Deeper areas of proteoglycan loss only, without chondrocyte loss or collagen disruption (reversible changes) may be present
- 4=marked degeneration, chondrocyte and proteoglycan loss with fibrillation extending through the mid zone through 3/4 f the depth (51-75% of cartilage thickness) but without complete (to the tidemark) loss of chondrocytes or proteoglycan, fibrillation/collagen damage generally extend through the mid zone (50% thickness) but deep zone collagen remains intact, or 51-75% overall chondrocyte and proteoglycan loss in the zone if lesion is focal and deeper in some areas. Deeper areas of proteoglycan loss only, without chondrocyte loss or collagen disruption (reversible changes) may be present
- 5=severe degeneration, chondrocyte and proteoglycan loss to near tidemark (76-100% of depth) with slightly less severe collagen damage or 76-100% overall chondrocyte and proteoglycan loss in the zone.

Each medial tibial plateau and medial femoral condyle anterior to posterior section was further divided visually with an ocular micrometer into fourths and numbered 1,2,3,4 with 1 being the outside and 4 being the inside quadrant. A mean score for the four levels was calculated. In addition, means of the 3 sections for each quadrant were determined in order to convey lesion severity in various axial to abaxial locations. Overall tibial and femoral values were summed for a total joint cartilage degeneration score.

Measurements of width of total lesion of any type (chondrocyte death, cloning, fibrillation, proteoglycan loss) were taken across the tibial and femoral surfaces to give a total lesion width. Significant lesion width (matrix loss extending beyond superficial degeneration; i.e., lesions consisting of changes greater than just proteoglycan loss) was also determined in an attempt to convey possible treatment differences based on evaluation of more serious and permanent/irreversible cartilage matrix changes.

Multiple depth measurements in areas of greatest lesion severity were taken for each of the 4 quadrants in each of the 3 anterior to posterior sections. These consisted of measurements taken from the surface (or estimated original surface when severe degenerative changes are present) to the point where cartilage appeared normal. A second measurement was taken in this same area to the depth of the tidemark so that the number expressed is a ratio of lesion depth vs. depth of tidemark. The mean values for each quadrant (medial to lateral) for the 3 anterior to posterior sections were determined as well as an overall mean for the joint. In this measurement, the minor changes consisting of proteoglycan loss only are not measured and only lesions with definite chondrocyte loss and sometimes fibrillation are included. This parameter should reflect a similar result to the significant width measurement as it is an indicator of beneficial effects on the more severe pathology.

Osteophytes for each compartment were measured (basal endochondral ossification to surface of cartilage cap) and severity grades assigned according to the following:

- none
- up to 1000 µm
- 1001 to 2000 µm
- 2001 or greater µm

**Sclerosis of subchondral bone**

Sclerosis of subchondral bone (an indication of altered load-bearing) was described and documented for femur using a comparison of medial to lateral, but was not included in the overall cartilage degeneration scores. This parameter was scored based on percent width of the medial femur with obviously thickened subchondral bone according to the following criteria:

- 0=normal
- 1=up to 10% of femur width has thickened trabeculae
- 2=11-30 % of femur width has thickened trabeculae
- 3=31-60 % of femur width has thickened trabeculae
- 4=61-90 % of femur width has thickened trabeculae
- 5=>90% of femur width has thickened trabeculae

An additional measure of bone area was performed as follows: The photo file was opened and a fixed segment of 1000 by 400 pixels was selected with the bottom left and right corners touching the tidemark:


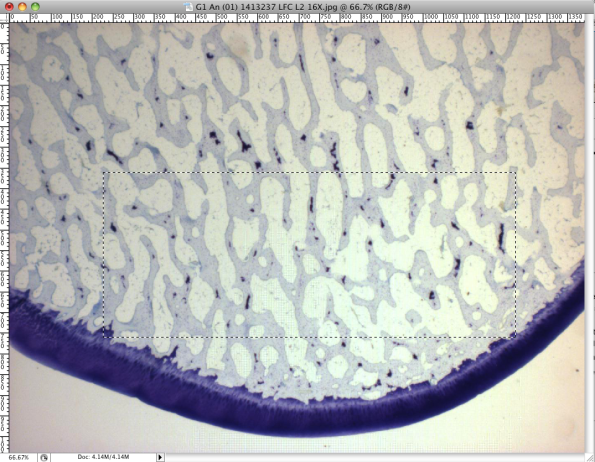


The selected segment was opened in a new file and segmented into fourths:


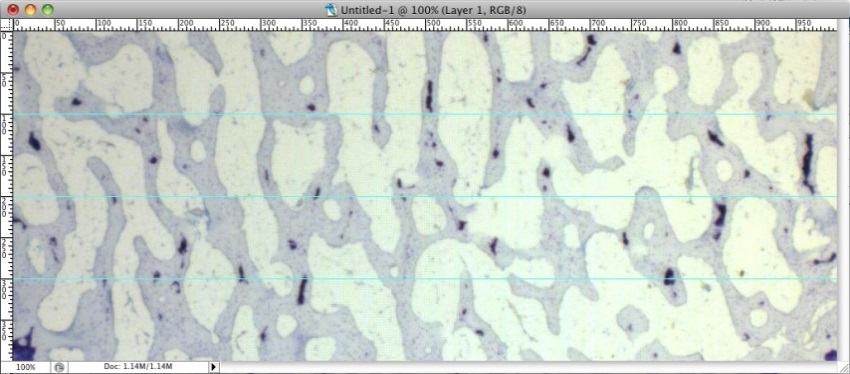


Black lines were drawn on the three segmenting lines in areas of no bone:


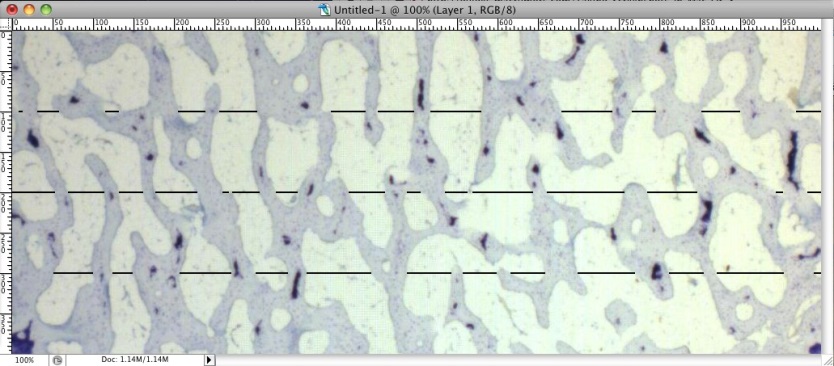


Lengths of each individual drawn segment were summed per line for a total length of area of no bone, then divided by the total length of the image and calculated into a percent. Percent of no bone was calculated for each of the three segmented lines and then averaged for a mean percent of no bone for the image.

**Collagen damage**

Collagen damage across the medial tibial plateau (most severely affected section) was quantified by measuring the total width of the following:

- Any damage (fibrillation ranging from superficial to full thickness loss)
- Severe damage (total or near total loss of collagen to tidemark, >90% thickness)
- Marked damage (extends through 61-90% of the cartilage thickness)
- Moderate damage (extends thru 31-60% of the cartilage thickness)
- Mild damage (extends through 11-30% of the cartilage thickness)
- Minimal damage (very superficial, affecting upper 10% only)

Single clefts were arbitrarily given a measure of 100 µm and the number of clefts was multiplied by 100 if several are present.

**Image analysis**

In order to quantitate and compare the cartilage matrix preservation, cartilage area measurements were taken from the most severely affected section of each animal. In this section, the most severely affected area - generally zone 2 - is analyzed at 25x magnification. Photomicrographs were taken with a digital microscope camera, and analyzed with appropriate software. The following measurements were taken:

- Total area from the tidemark to the surface (or projected surface in degenerated areas), extending from the axial to abaxial aspects of the medial tibial plateau zones in the most severely affected area (generally zone 2)
- Area of non-viable matrix (cartilage with less than 50% chondrocytes, proteoglycan, and intact collagen) and no matrix within the total area of zone 2 or most severely affected area in the photomicrograph
- Area of no matrix within the total area being analyzed

The area of non-viable matrix in zone under analysis was subtracted from the total area in the photomicrograph to get the area of viable matrix in this area, and the area of no matrix was subtracted from the total area to get the area of any matrix (collagen matrix with or without chondrocytes and proteoglycan). These two values were then compared back to the total area to derive the % viable area and the % any matrix in this area of greatest lesion severity, which were compared between groups.

All collected tissues from all animals were examined microscopically by a board certified veterinary pathologist (Dr. Alison Bendele) and observations were entered into a computer- assisted data retrieval system.
