# Supplementary material for: The selective cathepsin K inhibitor MIV-711 attenuates joint pathology in experimental animal models of osteoarthritis
Source: J Transl Med. 2018 Mar 9;16:56. doi: 10.1186/s12967-018-1425-7 (PMC5845353; doi:10.1186/s12967-018-1425-7)
Supplement: Supplementary file 3 — Additional file 3: Table S2. Rabbit anterior cruciate ligament transection model - histomorphometry data summary. [file 12967_2018_1425_MOESM3_ESM.docx]

**Additional file 3: Table S2**

**Rabbit anterior cruciate ligament transection model - Histomorphometric analysis**

The results of the histomorphometric analysis of bone and cartilage parameters are listed in the Tables S2a-h. Analysis was performed on all four condyles at two levels (Level 1 and 2) for each condyle:

- Lateral femoral condyle (LFC)
- Lateral tibial condyle (LTC)
- Medial femoral condyle (MFC)
- Medial tibial condyle (MTC)

The parameters assessed were the following:

- Bone Volume/Total Volume (BV/TV)
- Trabecular thickness (Tb.Th)
- Trabecular spacing (Tb.Sp)
- Trabecular number (Tb.N)
- Osteophyte area (Ophte.Ar)
- Cartilage area (Cart. Ar)
- Cartilage perimeter (Cart.Pm)
- Cartilage boundary (Cart.Bd)
- Cartilage width (Cart.Wi)
- Subchondral bone width (SCB.Wi)

The results are listed per group according to the following:

Group 1) sham + vehicle; Group 2) ACLT + vehicle; Group 3) ACLT + MIV-711, 30 µmol/kg (low dose) and Group 4) ACLT + MIV-711, 100 µmol/kg (high dose).

The statistical analyses in Tables S2a-h refer to the Shapiro-Wilk test (S-W) for normality and Levene’s test for homogeneity of variance. For data that passed both tests, a one-way ANOVA was performed, indicated as overall p value in Tables S2a-S2h. If the overall treatment effect was significant by ANOVA, a Dunnett’s test was performed to compare each treatment group compared to the control group (Group 2; ACLT + vehicle). The result of the Dunnett’s test is indicated in the last column of each table.

Table S2a. Histomorphometry Data Summary – Level 1 – LFC

| **Variable: BV/TV** (%) | | | | Levene: ns Overall p value: ns | | | |
| --- | --- | --- | --- | --- | --- | --- | --- |
| **Group** | **Mean** | **Median** | **SD** | **SEM** | **n** | **S-W** | **vs 2** |
| 1 | 42.60 | 43.34 | 5.79 | 2.36 | 6 | ns | ns |
| 2 | 37.65 | 36.81 | 3.41 | 1.29 | 7 | ns | na |
| 3 | 37.49 | 36.26 | 3.23 | 1.22 | 7 | ns | ns |
| 4 | 40.76 | 39.99 | 6.66 | 2.52 | 7 | ns | ns |

| **Variable: Tb.Th** (µm) | | | | Levene: ns  Overall p value: ns | | | |
| --- | --- | --- | --- | --- | --- | --- | --- |
| **Group** | **Mean** | **Median** | **SD** | **SEM** | **n** | **S-W** | **vs 2** |
| 1 | 148.05 | 144.85 | 18.60 | 7.59 | 6 | ns | ns |
| 2 | 135.75 | 138.76 | 21.54 | 8.14 | 7 | ns | na |
| 3 | 131.58 | 132.19 | 12.60 | 4.76 | 7 | ns | ns |
| 4 | 147.91 | 146.15 | 23.77 | 8.98 | 7 | ns | ns |

| **Variable: Tb.Sp** (µm) | | | | Levene: ns Overall p value: ns | | | |
| --- | --- | --- | --- | --- | --- | --- | --- |
| **Group** | **Mean** | **Median** | **SD** | **SEM** | **n** | **S-W** | **vs 2** |
| 1 | 201.83 | 200.25 | 37.38 | 15.26 | 6 | ns | ns |
| 2 | 223.54 | 226.57 | 18.86 | 7.13 | 7 | ns | na |
| 3 | 221.27 | 221.54 | 34.12 | 12.90 | 7 | ns | ns |
| 4 | 215.44 | 222.15 | 29.60 | 11.19 | 7 | ns | ns |

| **Variable: Tb.N** (1/mm) | | | | Levene: ns Overall p value: ns | | | |
| --- | --- | --- | --- | --- | --- | --- | --- |
| **Group** | **Mean** | **Median** | **SD** | **SEM** | **n** | **S-W** | **vs 2** |
| 1 | 2.89 | 2.92 | 0.32 | 0.13 | 6 | ns | ns |
| 2 | 2.81 | 2.71 | 0.29 | 0.11 | 7 | ns | na |
| 3 | 2.87 | 2.78 | 0.35 | 0.13 | 7 | ns | ns |
| 4 | 2.76 | 2.79 | 0.17 | 0.07 | 7 | ns | ns |

| **Variable: Ophte.Ar** (mm2) | | | | Levene: ns Overall p value: <.05 | | | |
| --- | --- | --- | --- | --- | --- | --- | --- |
| **Group** | **Mean** | **Median** | **SD** | **SEM** | **n** | **S-W** | **vs 2** |
| 1 | 0.04 | 0.00 | 0.10 | 0.04 | 6 | <.05 | **<.05** |
| 2 | 1.42 | 1.05 | 1.22 | 0.46 | 7 | ns | na |
| 3 | 2.48 | 1.89 | 2.28 | 0.86 | 7 | <.05 | ns |
| 4 | 3.18 | 1.06 | 6.27 | 2.37 | 7 | <.05 | ns |

Table S2a. Histomorphometry Data Summary – Level 1 – LFC (continued)

| **Variable: Cart.Ar** (mm2) | | | | Levene: ns Overall p value: <.05 | | | |
| --- | --- | --- | --- | --- | --- | --- | --- |
| **Group** | **Mean** | **Median** | **SD** | **SEM** | **n** | **S-W** | **vs 2** |
| 1 | 2.81 | 2.89 | 0.49 | 0.20 | 6 | ns | ns |
| 2 | 2.61 | 2.65 | 0.45 | 0.17 | 7 | ns | na |
| 3 | 2.52 | 2.55 | 0.34 | 0.13 | 7 | ns | ns |
| 4 | 3.28 | 3.33 | 0.47 | 0.18 | 7 | ns | **<.05** |

| **Variable: Cart.Pm** (mm) | | | | Levene: ns  Overall p value: <.05 | | | |
| --- | --- | --- | --- | --- | --- | --- | --- |
| **Group** | **Mean** | **Median** | **SD** | **SEM** | **n** | **S-W** | **vs 2** |
| 1 | 11.93 | 11.87 | 0.80 | 0.33 | 6 | ns | ns |
| 2 | 10.62 | 10.35 | 1.48 | 0.56 | 7 | ns | na |
| 3 | 10.23 | 10.40 | 1.26 | 0.48 | 7 | ns | ns |
| 4 | 11.98 | 12.38 | 1.15 | 0.43 | 7 | <.05 | ns |

| **Variable: Cart.Bd** (mm) | | | | Levene: ns Overall p value: <.05 | | | |
| --- | --- | --- | --- | --- | --- | --- | --- |
| **Group** | **Mean** | **Median** | **SD** | **SEM** | **n** | **S-W** | **vs 2** |
| 1 | 11.12 | 11.05 | 0.71 | 0.29 | 6 | ns | ns |
| 2 | 9.79 | 9.65 | 1.54 | 0.58 | 7 | ns | na |
| 3 | 9.42 | 9.52 | 1.22 | 0.46 | 7 | ns | ns |
| 4 | 11.05 | 11.43 | 1.06 | 0.40 | 7 | ns | ns |

| **Variable: Cart.Wi** (µm) | | | | Levene: ns Overall p value: ns | | | |
| --- | --- | --- | --- | --- | --- | --- | --- |
| **Group** | **Mean** | **Median** | **SD** | **SEM** | **n** | **S-W** | **vs 2** |
| 1 | 248.77 | 252.23 | 36.89 | 15.06 | 6 | ns | ns |
| 2 | 263.14 | 256.82 | 39.25 | 14.84 | 7 | ns | na |
| 3 | 263.55 | 269.31 | 31.57 | 11.93 | 7 | ns | ns |
| 4 | 290.77 | 272.05 | 36.50 | 13.79 | 7 | ns | ns |

| **Variable: SCB.Wi** (µm) | | | | Levene: ns Overall p value: ns | | | |
| --- | --- | --- | --- | --- | --- | --- | --- |
| **Group** | **Mean** | **Median** | **SD** | **SEM** | **n** | **S-W** | **vs 2** |
| 1 | 479.16 | 461.18 | 65.85 | 26.88 | 6 | ns | ns |
| 2 | 449.17 | 481.49 | 76.16 | 28.79 | 7 | ns | na |
| 3 | 527.88 | 537.74 | 76.16 | 28.79 | 7 | ns | ns |
| 4 | 478.03 | 466.56 | 52.92 | 20.00 | 7 | ns | ns |

Table S2b. Histomorphometry Data Summary – Level 1 – LTP

| **Variable: BV/TV** (%) | | | | Levene: ns Overall p value: ns | | | |
| --- | --- | --- | --- | --- | --- | --- | --- |
| **Group** | **Mean** | **Median** | **SD** | **SEM** | **n** | **S-W** | **vs 2** |
| 1 | 43.86 | 41.72 | 8.72 | 3.56 | 6 | ns | ns |
| 2 | 35.71 | 38.29 | 5.85 | 2.21 | 7 | <.05 | na |
| 3 | 43.89 | 43.62 | 6.56 | 2.48 | 7 | ns | ns |
| 4 | 43.87 | 45.02 | 3.59 | 1.36 | 7 | ns | ns |

| **Variable: Tb.Th** (µm) | | | | Levene: ns  Overall p value: <.05 | | | |
| --- | --- | --- | --- | --- | --- | --- | --- |
| **Group** | **Mean** | **Median** | **SD** | **SEM** | **n** | **S-W** | **vs 2** |
| 1 | 192.92 | 189.39 | 19.43 | 7.93 | 6 | ns | **<.05** |
| 2 | 148.07 | 138.90 | 19.32 | 7.30 | 7 | ns | na |
| 3 | 159.11 | 141.92 | 31.10 | 11.76 | 7 | ns | ns |
| 4 | 179.81 | 179.09 | 22.23 | 8.40 | 7 | ns | ns |

| **Variable: Tb.Sp** (µm) | | | | Levene: ns  Overall p value: ns | | | |
| --- | --- | --- | --- | --- | --- | --- | --- |
| **Group** | **Mean** | **Median** | **SD** | **SEM** | **n** | **S-W** | **vs 2** |
| 1 | 254.07 | 258.77 | 62.30 | 25.43 | 6 | ns | ns |
| 2 | 272.13 | 246.17 | 57.82 | 21.85 | 7 | ns | na |
| 3 | 202.57 | 211.29 | 28.41 | 10.74 | 7 | ns | ns |
| 4 | 232.42 | 223.55 | 43.91 | 16.60 | 7 | ns | ns |

| **Variable: Tb.N** (1/mm) | | | | Levene: ns Overall p value: <.05 | | | |
| --- | --- | --- | --- | --- | --- | --- | --- |
| **Group** | **Mean** | **Median** | **SD** | **SEM** | **n** | **S-W** | **vs 2** |
| 1 | 2.26 | 2.25 | 0.25 | 0.10 | 6 | ns | ns |
| 2 | 2.42 | 2.39 | 0.32 | 0.12 | 7 | ns | na |
| 3 | 2.79 | 2.77 | 0.28 | 0.11 | 7 | ns | ns |
| 4 | 2.47 | 2.39 | 0.38 | 0.14 | 7 | ns | ns |

| **Variable: Ophte.Ar** (mm2) | | | | Levene: ns  Overall p value: <.05 | | | |
| --- | --- | --- | --- | --- | --- | --- | --- |
| **Group** | **Mean** | **Median** | **SD** | **SEM** | **n** | **S-W** | **vs 2** |
| 1 | 0.02 | 0.00 | 0.05 | 0.02 | 6 | <.05 | **<.05** |
| 2 | 0.75 | 0.80 | 0.44 | 0.17 | 7 | ns | na |
| 3 | 0.43 | 0.58 | 0.31 | 0.12 | 7 | ns | ns |
| 4 | 0.62 | 0.37 | 0.53 | 0.20 | 7 | ns | ns |

Table S2b. Histomorphometry Data Summary – Level 1 – LTP (continued)

| **Variable: Cart.Ar** (mm2) | | | | Levene: ns Overall p value: ns | | | |
| --- | --- | --- | --- | --- | --- | --- | --- |
| **Group** | **Mean** | **Median** | **SD** | **SEM** | **n** | **S-W** | **vs 2** |
| 1 | 2.16 | 2.01 | 0.60 | 0.24 | 6 | ns | ns |
| 2 | 2.39 | 2.40 | 0.87 | 0.33 | 7 | ns | na |
| 3 | 2.03 | 2.02 | 0.43 | 0.16 | 7 | ns | ns |
| 4 | 2.49 | 2.63 | 0.62 | 0.23 | 7 | ns | ns |

| **Variable: Cart.Pm** (mm) | | | | Levene: ns  Overall p value: ns | | | |
| --- | --- | --- | --- | --- | --- | --- | --- |
| **Group** | **Mean** | **Median** | **SD** | **SEM** | **n** | **S-W** | **vs 2** |
| 1 | 6.33 | 6.06 | 0.74 | 0.30 | 6 | ns | ns |
| 2 | 5.91 | 6.19 | 0.96 | 0.36 | 7 | ns | na |
| 3 | 6.15 | 6.13 | 0.39 | 0.15 | 7 | ns | ns |
| 4 | 6.48 | 6.62 | 0.40 | 0.15 | 7 | ns | ns |

| **Variable: Cart.Bd** (mm) | | | | Levene: ns Overall p value: ns | | | |
| --- | --- | --- | --- | --- | --- | --- | --- |
| **Group** | **Mean** | **Median** | **SD** | **SEM** | **n** | **S-W** | **vs 2** |
| 1 | 6.38 | 6.10 | 0.75 | 0.31 | 6 | ns | ns |
| 2 | 5.94 | 6.36 | 1.00 | 0.38 | 7 | ns | na |
| 3 | 6.10 | 6.13 | 0.38 | 0.14 | 7 | ns | ns |
| 4 | 6.56 | 6.67 | 0.44 | 0.17 | 7 | ns | ns |

| **Variable: Cart.Wi** (µm) | | | | Levene: ns Overall p value: ns | | | |
| --- | --- | --- | --- | --- | --- | --- | --- |
| **Group** | **Mean** | **Median** | **SD** | **SEM** | **n** | **S-W** | **vs 2** |
| 1 | 340.34 | 340.38 | 64.66 | 26.40 | 6 | ns | ns |
| 2 | 406.56 | 427.00 | 99.39 | 37.56 | 7 | ns | na |
| 3 | 340.85 | 342.70 | 69.09 | 26.11 | 7 | ns | ns |
| 4 | 391.09 | 393.49 | 86.78 | 32.80 | 7 | ns | ns |

| **Variable: SCB.Wi** (µm) | | | | Levene: ns Overall p value: ns | | | |
| --- | --- | --- | --- | --- | --- | --- | --- |
| **Group** | **Mean** | **Median** | **SD** | **SEM** | **n** | **S-W** | **vs 2** |
| 1 | 560.15 | 591.34 | 87.97 | 35.92 | 6 | ns | ns |
| 2 | 472.20 | 426.04 | 135.72 | 51.30 | 7 | ns | na |
| 3 | 532.09 | 572.30 | 101.64 | 38.42 | 7 | ns | ns |
| 4 | 557.78 | 592.81 | 71.69 | 27.10 | 7 | <.05 | ns |

Table S2c. Histomorphometry Data Summary – Level 1 – MFC

| **Variable: BV/TV** (%) | | | | Levene: ns Overall p value: ns | | | |
| --- | --- | --- | --- | --- | --- | --- | --- |
| **Group** | **Mean** | **Median** | **SD** | **SEM** | **n** | **S-W** | **vs 2** |
| 1 | 49.52 | 50.77 | 4.92 | 2.01 | 6 | ns | ns |
| 2 | 46.84 | 47.76 | 4.99 | 1.89 | 7 | ns | na |
| 3 | 52.87 | 51.00 | 7.99 | 3.02 | 7 | ns | ns |
| 4 | 46.41 | 48.01 | 7.01 | 2.65 | 7 | <.05 | ns |

| **Variable: Tb.Th** (µm) | | | | Levene: ns  Overall p value: ns | | | |
| --- | --- | --- | --- | --- | --- | --- | --- |
| **Group** | **Mean** | **Median** | **SD** | **SEM** | **n** | **S-W** | **vs 2** |
| 1 | 166.78 | 163.66 | 12.28 | 5.01 | 6 | ns | ns |
| 2 | 162.68 | 165.62 | 14.07 | 5.32 | 7 | ns | na |
| 3 | 190.82 | 169.90 | 57.09 | 21.58 | 7 | ns | ns |
| 4 | 161.31 | 161.50 | 18.91 | 7.15 | 7 | ns | ns |

| **Variable: Tb.Sp** (µm) | | | | Levene: ns Overall p value: ns | | | |
| --- | --- | --- | --- | --- | --- | --- | --- |
| **Group** | **Mean** | **Median** | **SD** | **SEM** | **n** | **S-W** | **vs 2** |
| 1 | 172.38 | 166.55 | 33.65 | 13.74 | 6 | ns | ns |
| 2 | 185.40 | 184.58 | 22.06 | 8.34 | 7 | ns | na |
| 3 | 164.11 | 161.32 | 14.34 | 5.42 | 7 | ns | ns |
| 4 | 189.94 | 173.69 | 45.95 | 17.37 | 7 | <.05 | ns |

| **Variable: Tb.N** (1/mm) | | | | Levene: ns Overall p value: ns | | | |
| --- | --- | --- | --- | --- | --- | --- | --- |
| **Group** | **Mean** | **Median** | **SD** | **SEM** | **n** | **S-W** | **vs 2** |
| 1 | 2.98 | 2.91 | 0.32 | 0.13 | 6 | ns | ns |
| 2 | 2.88 | 2.86 | 0.09 | 0.04 | 7 | ns | na |
| 3 | 2.87 | 2.98 | 0.40 | 0.15 | 7 | ns | ns |
| 4 | 2.87 | 2.92 | 0.28 | 0.10 | 7 | ns | ns |

| **Variable: Ophte.Ar** (mm2) | | | | Levene: ns Overall p value: <.05 | | | |
| --- | --- | --- | --- | --- | --- | --- | --- |
| **Group** | **Mean** | **Median** | **SD** | **SEM** | **n** | **S-W** | **vs 2** |
| 1 | 0.02 | 0.00 | 0.05 | 0.02 | 6 | <.05 | **<.05** |
| 2 | 2.01 | 2.05 | 1.21 | 0.46 | 7 | ns | na |
| 3 | 2.75 | 2.12 | 2.41 | 0.91 | 7 | ns | ns |
| 4 | 5.87 | 1.02 | 9.53 | 3.60 | 7 | <.05 | ns |

Table S2c. Histomorphometry Data Summary – Level 1 – MFC (continued)

| **Variable: Cart.Ar** (mm2) | | | | Levene: ns Overall p value: ns | | | |
| --- | --- | --- | --- | --- | --- | --- | --- |
| **Group** | **Mean** | **Median** | **SD** | **SEM** | **n** | **S-W** | **vs 2** |
| 1 | 2.74 | 2.64 | 0.99 | 0.40 | 6 | ns | ns |
| 2 | 3.32 | 2.90 | 0.78 | 0.29 | 7 | ns | na |
| 3 | 2.30 | 2.03 | 1.01 | 0.38 | 7 | ns | ns |
| 4 | 2.99 | 3.31 | 0.70 | 0.26 | 7 | ns | ns |

| **Variable: Cart.Pm** (mm) | | | | Levene: ns  Overall p value: <.05 | | | |
| --- | --- | --- | --- | --- | --- | --- | --- |
| **Group** | **Mean** | **Median** | **SD** | **SEM** | **n** | **S-W** | **vs 2** |
| 1 | 9.10 | 9.03 | 0.88 | 0.36 | 6 | ns | ns |
| 2 | 10.07 | 9.84 | 0.64 | 0.24 | 7 | ns | na |
| 3 | 9.31 | 9.55 | 0.79 | 0.30 | 7 | ns | ns |
| 4 | 10.24 | 10.20 | 0.75 | 0.28 | 7 | ns | ns |

| **Variable: Cart.Bd** (mm) | | | | Levene: ns Overall p value: <.05 | | | |
| --- | --- | --- | --- | --- | --- | --- | --- |
| **Group** | **Mean** | **Median** | **SD** | **SEM** | **n** | **S-W** | **vs 2** |
| 1 | 8.22 | 8.18 | 0.56 | 0.23 | 6 | ns | ns |
| 2 | 8.94 | 8.64 | 0.53 | 0.20 | 7 | ns | na |
| 3 | 8.37 | 8.42 | 0.61 | 0.23 | 7 | ns | ns |
| 4 | 8.94 | 8.89 | 0.34 | 0.13 | 7 | ns | ns |

| **Variable: Cart.Wi** (µm) | | | | Levene: ns Overall p value: ns | | | |
| --- | --- | --- | --- | --- | --- | --- | --- |
| **Group** | **Mean** | **Median** | **SD** | **SEM** | **n** | **S-W** | **vs 2** |
| 1 | 320.89 | 328.81 | 92.78 | 37.88 | 6 | ns | ns |
| 2 | 354.77 | 316.61 | 82.86 | 31.32 | 7 | <.05 | na |
| 3 | 264.49 | 233.46 | 101.55 | 38.38 | 7 | ns | ns |
| 4 | 323.96 | 341.58 | 74.09 | 28.00 | 7 | ns | ns |

| **Variable: SCB.Wi** (µm) | | | | Levene: ns Overall p value: <.05 | | | |
| --- | --- | --- | --- | --- | --- | --- | --- |
| **Group** | **Mean** | **Median** | **SD** | **SEM** | **n** | **S-W** | **vs 2** |
| 1 | 534.82 | 572.11 | 85.50 | 34.90 | 6 | ns | ns |
| 2 | 562.50 | 540.74 | 94.82 | 35.84 | 7 | ns | na |
| 3 | 796.52 | 695.73 | 233.56 | 88.28 | 7 | ns | **<.05** |
| 4 | 655.48 | 627.36 | 121.26 | 45.83 | 7 | ns | ns |

Table S2d. Histomorphometry Data Summary – Level 1 – MTP

| **Variable: BV/TV** (%) | | | | Levene: ns Overall p value: ns | | | |
| --- | --- | --- | --- | --- | --- | --- | --- |
| **Group** | **Mean** | **Median** | **SD** | **SEM** | **n** | **S-W** | **vs 2** |
| 1 | 38.68 | 39.71 | 6.76 | 2.76 | 6 | ns | ns |
| 2 | 29.61 | 30.09 | 7.43 | 2.81 | 7 | ns | na |
| 3 | 37.43 | 39.13 | 6.94 | 2.62 | 7 | ns | ns |
| 4 | 35.92 | 35.01 | 4.18 | 1.58 | 7 | ns | ns |

| **Variable: Tb.Th** (µm) | | | | Levene: ns  Overall p value: <.05 | | | |
| --- | --- | --- | --- | --- | --- | --- | --- |
| **Group** | **Mean** | **Median** | **SD** | **SEM** | **n** | **S-W** | **vs 2** |
| 1 | 223.69 | 244.98 | 38.75 | 15.82 | 6 | <.05 | **<.05** |
| 2 | 149.20 | 156.32 | 27.03 | 10.22 | 7 | ns | na |
| 3 | 182.44 | 174.84 | 37.93 | 14.34 | 7 | ns | ns |
| 4 | 190.56 | 188.86 | 20.76 | 7.85 | 7 | ns | ns |

| **Variable: Tb.Sp** (µm) | | | | Levene: ns Overall p value: ns | | | |
| --- | --- | --- | --- | --- | --- | --- | --- |
| **Group** | **Mean** | **Median** | **SD** | **SEM** | **n** | **S-W** | **vs 2** |
| 1 | 359.29 | 337.04 | 74.11 | 30.25 | 6 | ns | ns |
| 2 | 366.38 | 339.27 | 85.96 | 32.49 | 7 | ns | na |
| 3 | 306.15 | 294.58 | 48.56 | 18.36 | 7 | ns | ns |
| 4 | 346.56 | 370.02 | 71.00 | 26.84 | 7 | ns | ns |

| **Variable: Tb.N** (1/mm) | | | | Levene: ns Overall p value: ns | | | |
| --- | --- | --- | --- | --- | --- | --- | --- |
| **Group** | **Mean** | **Median** | **SD** | **SEM** | **n** | **S-W** | **vs 2** |
| 1 | 1.74 | 1.72 | 0.24 | 0.10 | 6 | ns | ns |
| 2 | 1.97 | 1.99 | 0.24 | 0.09 | 7 | ns | na |
| 3 | 2.07 | 1.97 | 0.22 | 0.08 | 7 | ns | ns |
| 4 | 1.91 | 1.80 | 0.35 | 0.13 | 7 | ns | ns |

| **Variable: Ophte.Ar** (mm2) | | | | Levene: <.05 Overall p value: <.05 | | | |
| --- | --- | --- | --- | --- | --- | --- | --- |
| **Group** | **Mean** | **Median** | **SD** | **SEM** | **n** | **S-W** | **vs 2** |
| 1 | 0.23 | 0.20 | 0.25 | 0.10 | 6 | <.05 | **<.05** |
| 2 | 4.69 | 5.15 | 3.58 | 1.35 | 7 | ns | na |
| 3 | 7.76 | 7.61 | 3.90 | 1.47 | 7 | ns | ns |
| 4 | 9.09 | 7.85 | 6.83 | 2.58 | 7 | ns | ns |

Table S2d. Histomorphometry Data Summary – Level 1 – MTP (continued)

| **Variable: Cart.Ar** (mm2) | | | | Levene: ns Overall p value: ns | | | |
| --- | --- | --- | --- | --- | --- | --- | --- |
| **Group** | **Mean** | **Median** | **SD** | **SEM** | **n** | **S-W** | **vs 2** |
| 1 | 3.93 | 3.90 | 0.80 | 0.33 | 6 | ns | ns |
| 2 | 3.15 | 3.58 | 1.19 | 0.45 | 7 | ns | na |
| 3 | 3.43 | 3.15 | 1.00 | 0.38 | 7 | ns | ns |
| 4 | 2.98 | 3.00 | 0.84 | 0.32 | 7 | ns | ns |

| **Variable: Cart.Pm** (mm) | | | | Levene: ns  Overall p value: ns | | | |
| --- | --- | --- | --- | --- | --- | --- | --- |
| **Group** | **Mean** | **Median** | **SD** | **SEM** | **n** | **S-W** | **vs 2** |
| 1 | 5.89 | 5.39 | 1.78 | 0.73 | 6 | ns | ns |
| 2 | 5.50 | 5.76 | 1.66 | 0.63 | 7 | ns | na |
| 3 | 5.96 | 5.73 | 0.72 | 0.27 | 7 | ns | ns |
| 4 | 6.31 | 5.70 | 2.17 | 0.82 | 7 | ns | ns |

| **Variable: Cart.Bd** (mm) | | | | Levene: ns Overall p value: ns | | | |
| --- | --- | --- | --- | --- | --- | --- | --- |
| **Group** | **Mean** | **Median** | **SD** | **SEM** | **n** | **S-W** | **vs 2** |
| 1 | 5.60 | 5.72 | 0.70 | 0.28 | 6 | ns | ns |
| 2 | 5.08 | 5.49 | 1.37 | 0.52 | 7 | ns | na |
| 3 | 5.74 | 5.82 | 0.61 | 0.23 | 7 | ns | ns |
| 4 | 5.22 | 5.08 | 0.58 | 0.22 | 7 | ns | ns |

| **Variable: Cart.Wi** (µm) | | | | Levene: ns Overall p value: ns | | | |
| --- | --- | --- | --- | --- | --- | --- | --- |
| **Group** | **Mean** | **Median** | **SD** | **SEM** | **n** | **S-W** | **vs 2** |
| 1 | 708.97 | 721.31 | 92.47 | 37.75 | 6 | ns | ns |
| 2 | 634.32 | 676.43 | 135.95 | 51.38 | 7 | <.05 | na |
| 3 | 627.75 | 633.18 | 147.01 | 55.56 | 7 | ns | ns |
| 4 | 618.52 | 551.09 | 141.95 | 53.65 | 7 | ns | ns |

| **Variable: SCB.Wi** (µm) | | | | Levene: ns Overall p value: ns | | | |
| --- | --- | --- | --- | --- | --- | --- | --- |
| **Group** | **Mean** | **Median** | **SD** | **SEM** | **n** | **S-W** | **vs 2** |
| 1 | 730.52 | 697.97 | 209.14 | 85.38 | 6 | ns | ns |
| 2 | 638.27 | 577.90 | 150.26 | 56.79 | 7 | ns | na |
| 3 | 718.50 | 715.91 | 79.74 | 30.14 | 7 | ns | ns |
| 4 | 839.92 | 790.92 | 120.02 | 45.36 | 7 | <.05 | ns |

Table S2e. Histomorphometry Data Summary – Level 2 – LFC

| **Variable: BV/TV** (%) | | | | Levene: ns Overall p value: ns | | | |
| --- | --- | --- | --- | --- | --- | --- | --- |
| **Group** | **Mean** | **Median** | **SD** | **SEM** | **n** | **S-W** | **vs 2** |
| 1 | 39.54 | 40.60 | 8.31 | 3.39 | 6 | ns | ns |
| 2 | 35.71 | 35.19 | 6.27 | 2.37 | 7 | ns | na |
| 3 | 36.23 | 35.15 | 3.70 | 1.40 | 7 | ns | ns |
| 4 | 36.75 | 38.04 | 4.70 | 1.78 | 7 | ns | ns |

| **Variable: Tb.Th** (µm) | | | | Levene: ns  Overall p value: ns | | | |
| --- | --- | --- | --- | --- | --- | --- | --- |
| **Group** | **Mean** | **Median** | **SD** | **SEM** | **n** | **S-W** | **vs 2** |
| 1 | 131.08 | 135.74 | 28.12 | 11.48 | 6 | ns | ns |
| 2 | 133.46 | 129.51 | 27.30 | 10.32 | 7 | ns | na |
| 3 | 129.54 | 124.26 | 25.64 | 9.69 | 7 | ns | ns |
| 4 | 120.73 | 120.14 | 7.59 | 2.87 | 7 | ns | ns |

| **Variable: Tb.Sp** (µm) | | | | Levene: ns Overall p value: ns | | | |
| --- | --- | --- | --- | --- | --- | --- | --- |
| **Group** | **Mean** | **Median** | **SD** | **SEM** | **n** | **S-W** | **vs 2** |
| 1 | 201.35 | 209.27 | 35.31 | 14.42 | 6 | ns | ns |
| 2 | 241.60 | 219.29 | 48.59 | 18.37 | 7 | ns | na |
| 3 | 227.32 | 224.93 | 37.07 | 14.01 | 7 | ns | ns |
| 4 | 212.50 | 210.44 | 44.77 | 16.92 | 7 | ns | ns |

| **Variable: Tb.N** (1/mm) | | | | Levene: ns Overall p value: ns | | | |
| --- | --- | --- | --- | --- | --- | --- | --- |
| **Group** | **Mean** | **Median** | **SD** | **SEM** | **n** | **S-W** | **vs 2** |
| 1 | 3.04 | 3.03 | 0.32 | 0.13 | 6 | ns | ns |
| 2 | 2.72 | 2.72 | 0.40 | 0.15 | 7 | ns | na |
| 3 | 2.86 | 3.01 | 0.42 | 0.16 | 7 | ns | ns |
| 4 | 3.05 | 2.97 | 0.45 | 0.17 | 7 | ns | ns |

| **Variable: Ophte.Ar** (mm2) | | | | Levene: ns Overall p value: <.05 | | | |
| --- | --- | --- | --- | --- | --- | --- | --- |
| **Group** | **Mean** | **Median** | **SD** | **SEM** | **n** | **S-W** | **vs 2** |
| 1 | 0.32 | 0.29 | 0.36 | 0.15 | 6 | <.05 | **<.05** |
| 2 | 2.44 | 2.33 | 1.19 | 0.45 | 7 | ns | na |
| 3 | 1.80 | 1.91 | 0.86 | 0.32 | 7 | ns | ns |
| 4 | 4.60 | 1.57 | 8.18 | 3.09 | 7 | <.05 | ns |

Table S2e. Histomorphometry Data Summary – Level 2 – LFC (continued)

| **Variable: Cart.Ar** (mm2) | | | | Levene: ns Overall p value: ns | | | |
| --- | --- | --- | --- | --- | --- | --- | --- |
| **Group** | **Mean** | **Median** | **SD** | **SEM** | **n** | **S-W** | **vs 2** |
| 1 | 1.51 | 1.36 | 0.37 | 0.15 | 6 | <.05 | ns |
| 2 | 1.45 | 1.46 | 0.33 | 0.12 | 7 | ns | na |
| 3 | 1.54 | 1.50 | 0.25 | 0.09 | 7 | ns | ns |
| 4 | 1.69 | 1.76 | 0.29 | 0.11 | 7 | <.05 | ns |

| **Variable: Cart.Pm** (mm) | | | | Levene: ns  Overall p value: <.05 | | | |
| --- | --- | --- | --- | --- | --- | --- | --- |
| **Group** | **Mean** | **Median** | **SD** | **SEM** | **n** | **S-W** | **vs 2** |
| 1 | 8.24 | 7.85 | 1.24 | 0.50 | 6 | <.05 | ns |
| 2 | 6.69 | 6.93 | 0.91 | 0.34 | 7 | ns | na |
| 3 | 6.68 | 6.07 | 1.14 | 0.43 | 7 | ns | ns |
| 4 | 8.13 | 8.16 | 0.38 | 0.14 | 7 | ns | **<.05** |

| **Variable: Cart.Bd** (mm) | | | | Levene: ns Overall p value: <.05 | | | |
| --- | --- | --- | --- | --- | --- | --- | --- |
| **Group** | **Mean** | **Median** | **SD** | **SEM** | **n** | **S-W** | **vs 2** |
| 1 | 7.68 | 7.43 | 1.08 | 0.44 | 6 | <.05 | **<.05** |
| 2 | 6.07 | 6.25 | 0.93 | 0.35 | 7 | ns | na |
| 3 | 5.95 | 5.45 | 1.18 | 0.45 | 7 | ns | ns |
| 4 | 7.36 | 7.45 | 0.42 | 0.16 | 7 | ns | **<.05** |

| **Variable: Cart.Wi** (µm) | | | | Levene: ns Overall p value: ns | | | |
| --- | --- | --- | --- | --- | --- | --- | --- |
| **Group** | **Mean** | **Median** | **SD** | **SEM** | **n** | **S-W** | **vs 2** |
| 1 | 191.20 | 189.20 | 14.34 | 5.85 | 6 | ns | ns |
| 2 | 228.48 | 218.55 | 51.30 | 19.39 | 7 | ns | na |
| 3 | 252.24 | 265.00 | 47.52 | 17.96 | 7 | ns | ns |
| 4 | 223.79 | 238.09 | 38.52 | 14.56 | 7 | <.05 | ns |

| **Variable: SCB.Wi** (µm) | | | | Levene: ns Overall p value: <.05 | | | |
| --- | --- | --- | --- | --- | --- | --- | --- |
| **Group** | **Mean** | **Median** | **SD** | **SEM** | **n** | **S-W** | **vs 2** |
| 1 | 462.72 | 453.32 | 53.44 | 21.82 | 6 | ns | ns |
| 2 | 538.33 | 519.14 | 121.42 | 45.89 | 7 | ns | na |
| 3 | 625.26 | 686.73 | 122.06 | 46.14 | 7 | ns | ns |
| 4 | 500.70 | 462.81 | 63.55 | 24.02 | 7 | ns | ns |

Table S2f. Histomorphometry Data Summary – Level 2 – LTP

| **Variable: BV/TV** (%) | | | | Levene: ns Overall p value: ns | | | |
| --- | --- | --- | --- | --- | --- | --- | --- |
| **Group** | **Mean** | **Median** | **SD** | **SEM** | **n** | **S-W** | **vs 2** |
| 1 | 37.00 | 38.65 | 8.52 | 3.48 | 6 | ns | ns |
| 2 | 36.27 | 36.34 | 5.33 | 2.01 | 7 | ns | na |
| 3 | 42.02 | 40.17 | 7.31 | 2.98 | 6 | ns | ns |
| 4 | 39.84 | 38.48 | 7.83 | 2.96 | 7 | ns | ns |

| **Variable: Tb.Th** (µm) | | | | Levene: ns  Overall p value: ns | | | |
| --- | --- | --- | --- | --- | --- | --- | --- |
| **Group** | **Mean** | **Median** | **SD** | **SEM** | **n** | **S-W** | **vs 2** |
| 1 | 148.07 | 150.06 | 23.70 | 9.67 | 6 | ns | ns |
| 2 | 133.48 | 131.28 | 23.96 | 9.05 | 7 | ns | na |
| 3 | 146.36 | 148.82 | 32.82 | 13.40 | 6 | ns | ns |
| 4 | 161.80 | 153.89 | 25.16 | 9.51 | 7 | ns | ns |

| **Variable: Tb.Sp** (µm) | | | | Levene: ns Overall p value: ns | | | |
| --- | --- | --- | --- | --- | --- | --- | --- |
| **Group** | **Mean** | **Median** | **SD** | **SEM** | **n** | **S-W** | **vs 2** |
| 1 | 266.11 | 218.03 | 97.74 | 39.90 | 6 | <.05 | ns |
| 2 | 234.83 | 231.38 | 36.54 | 13.81 | 7 | ns | na |
| 3 | 202.17 | 206.70 | 41.66 | 17.01 | 6 | ns | ns |
| 4 | 250.77 | 246.02 | 62.73 | 23.71 | 7 | ns | ns |

| **Variable: Tb.N** (1/mm) | | | | Levene: ns Overall p value: ns | | | |
| --- | --- | --- | --- | --- | --- | --- | --- |
| **Group** | **Mean** | **Median** | **SD** | **SEM** | **n** | **S-W** | **vs 2** |
| 1 | 2.51 | 2.56 | 0.50 | 0.20 | 6 | ns | ns |
| 2 | 2.75 | 2.78 | 0.31 | 0.12 | 7 | ns | na |
| 3 | 2.93 | 3.04 | 0.43 | 0.18 | 6 | ns | ns |
| 4 | 2.47 | 2.50 | 0.36 | 0.13 | 7 | ns | ns |

| **Variable: Ophte.Ar** (mm2) | | | | Levene: <.05 Overall p value: <.05 | | | |
| --- | --- | --- | --- | --- | --- | --- | --- |
| **Group** | **Mean** | **Median** | **SD** | **SEM** | **n** | **S-W** | **vs 2** |
| 1 | 0.00 | 0.00 | 0.00 | 0.00 | 6 | ns | **<.05** |
| 2 | 1.57 | 1.56 | 0.51 | 0.19 | 7 | ns | na |
| 3 | 1.40 | 1.23 | 0.84 | 0.34 | 6 | ns | ns |
| 4 | 1.37 | 1.37 | 0.49 | 0.19 | 7 | ns | ns |

Table S2f. Histomorphometry Data Summary – Level 2 – LTP (continued)

| **Variable: Cart.Ar** (mm2) | | | | Levene: ns Overall p value: ns | | | |
| --- | --- | --- | --- | --- | --- | --- | --- |
| **Group** | **Mean** | **Median** | **SD** | **SEM** | **n** | **S-W** | **vs 2** |
| 1 | 0.81 | 0.87 | 0.32 | 0.13 | 6 | ns | ns |
| 2 | 0.64 | 0.50 | 0.34 | 0.13 | 7 | ns | na |
| 3 | 0.88 | 0.95 | 0.27 | 0.11 | 6 | ns | ns |
| 4 | 1.20 | 1.00 | 0.54 | 0.21 | 7 | ns | ns |

| **Variable: Cart.Pm** (mm) | | | | Levene: ns  Overall p value: ns | | | |
| --- | --- | --- | --- | --- | --- | --- | --- |
| **Group** | **Mean** | **Median** | **SD** | **SEM** | **n** | **S-W** | **vs 2** |
| 1 | 4.53 | 4.72 | 1.26 | 0.52 | 6 | ns | ns |
| 2 | 3.47 | 3.73 | 1.09 | 0.41 | 7 | ns | na |
| 3 | 4.26 | 4.48 | 0.85 | 0.35 | 6 | ns | ns |
| 4 | 5.10 | 4.58 | 1.77 | 0.67 | 7 | ns | ns |

| **Variable: Cart.Bd** (mm) | | | | Levene: ns Overall p value: ns | | | |
| --- | --- | --- | --- | --- | --- | --- | --- |
| **Group** | **Mean** | **Median** | **SD** | **SEM** | **n** | **S-W** | **vs 2** |
| 1 | 4.57 | 4.76 | 1.19 | 0.48 | 6 | ns | ns |
| 2 | 3.45 | 3.75 | 1.08 | 0.41 | 7 | ns | na |
| 3 | 4.27 | 4.41 | 0.77 | 0.31 | 6 | ns | ns |
| 4 | 4.73 | 4.37 | 1.19 | 0.45 | 7 | ns | ns |

| **Variable: Cart.Wi** (µm) | | | | Levene: ns Overall p value: ns | | | |
| --- | --- | --- | --- | --- | --- | --- | --- |
| **Group** | **Mean** | **Median** | **SD** | **SEM** | **n** | **S-W** | **vs 2** |
| 1 | 188.93 | 185.69 | 51.30 | 20.94 | 6 | ns | ns |
| 2 | 185.85 | 160.21 | 69.82 | 26.39 | 7 | ns | na |
| 3 | 207.98 | 203.75 | 34.83 | 14.22 | 6 | ns | ns |
| 4 | 263.50 | 255.71 | 84.78 | 32.04 | 7 | ns | ns |

| **Variable: SCB.Wi** (µm) | | | | Levene: ns Overall p value: ns | | | |
| --- | --- | --- | --- | --- | --- | --- | --- |
| **Group** | **Mean** | **Median** | **SD** | **SEM** | **n** | **S-W** | **vs 2** |
| 1 | 566.07 | 569.21 | 54.53 | 22.26 | 6 | ns | ns |
| 2 | 458.64 | 397.65 | 133.79 | 50.57 | 7 | ns | na |
| 3 | 531.17 | 547.82 | 82.53 | 33.69 | 6 | ns | ns |
| 4 | 559.05 | 594.94 | 124.81 | 47.17 | 7 | ns | ns |

Table S2g. Histomorphometry Data Summary – Level 2 – MFC

| **Variable: BV/TV** (%) | | | | Levene: ns Overall p value: ns | | | |
| --- | --- | --- | --- | --- | --- | --- | --- |
| **Group** | **Mean** | **Median** | **SD** | **SEM** | **n** | **S-W** | **vs 2** |
| 1 | 42.79 | 41.49 | 6.09 | 2.48 | 6 | ns | ns |
| 2 | 44.40 | 44.87 | 6.19 | 2.34 | 7 | ns | na |
| 3 | 50.84 | 46.53 | 11.82 | 4.47 | 7 | ns | ns |
| 4 | 43.61 | 41.52 | 8.69 | 3.28 | 7 | ns | ns |

| **Variable: Tb.Th** (µm) | | | | Levene: <.05  Overall p value: ns | | | |
| --- | --- | --- | --- | --- | --- | --- | --- |
| **Group** | **Mean** | **Median** | **SD** | **SEM** | **n** | **S-W** | **vs 2** |
| 1 | 144.80 | 143.46 | 11.52 | 4.70 | 6 | ns | ns |
| 2 | 153.43 | 153.20 | 19.02 | 7.19 | 7 | ns | na |
| 3 | 189.67 | 161.11 | 68.57 | 25.92 | 7 | ns | ns |
| 4 | 144.65 | 140.49 | 25.02 | 9.46 | 7 | ns | ns |

| **Variable: Tb.Sp** (µm) | | | | Levene: ns Overall p value: ns | | | |
| --- | --- | --- | --- | --- | --- | --- | --- |
| **Group** | **Mean** | **Median** | **SD** | **SEM** | **n** | **S-W** | **vs 2** |
| 1 | 196.30 | 199.85 | 33.75 | 13.78 | 6 | ns | ns |
| 2 | 193.09 | 188.22 | 27.21 | 10.29 | 7 | ns | na |
| 3 | 174.68 | 185.17 | 29.00 | 10.96 | 7 | ns | ns |
| 4 | 189.22 | 197.91 | 38.72 | 14.63 | 7 | ns | ns |

| **Variable: Tb.N** (1/mm) | | | | Levene: ns Overall p value: ns | | | |
| --- | --- | --- | --- | --- | --- | --- | --- |
| **Group** | **Mean** | **Median** | **SD** | **SEM** | **n** | **S-W** | **vs 2** |
| 1 | 2.95 | 2.92 | 0.23 | 0.10 | 6 | ns | ns |
| 2 | 2.89 | 2.90 | 0.11 | 0.04 | 7 | ns | na |
| 3 | 2.80 | 2.86 | 0.45 | 0.17 | 7 | ns | ns |
| 4 | 3.01 | 3.07 | 0.25 | 0.09 | 7 | ns | ns |

| **Variable: Ophte.Ar** (mm2) | | | | Levene: ns Overall p value: <.05 | | | |
| --- | --- | --- | --- | --- | --- | --- | --- |
| **Group** | **Mean** | **Median** | **SD** | **SEM** | **n** | **S-W** | **vs 2** |
| 1 | 0.18 | 0.00 | 0.44 | 0.18 | 6 | <.05 | **<.05** |
| 2 | 2.14 | 2.35 | 1.00 | 0.38 | 7 | <.05 | na |
| 3 | 2.89 | 3.00 | 1.95 | 0.74 | 7 | ns | ns |
| 4 | 2.78 | 2.43 | 0.93 | 0.35 | 7 | ns | ns |

Table S2g. Histomorphometry Data Summary – Level 2 – MFC (continued)

| **Variable: Cart.Ar** (mm2) | | | | Levene: ns Overall p value: ns | | | |
| --- | --- | --- | --- | --- | --- | --- | --- |
| **Group** | **Mean** | **Median** | **SD** | **SEM** | **n** | **S-W** | **vs 2** |
| 1 | 1.72 | 1.60 | 0.39 | 0.16 | 6 | ns | ns |
| 2 | 1.75 | 1.81 | 0.35 | 0.13 | 7 | ns | na |
| 3 | 1.47 | 1.46 | 0.33 | 0.12 | 7 | ns | ns |
| 4 | 1.41 | 1.41 | 0.28 | 0.10 | 7 | ns | ns |

| **Variable: Cart.Pm** (mm) | | | | Levene: ns  Overall p value: ns | | | |
| --- | --- | --- | --- | --- | --- | --- | --- |
| **Group** | **Mean** | **Median** | **SD** | **SEM** | **n** | **S-W** | **vs 2** |
| 1 | 7.70 | 7.35 | 0.89 | 0.36 | 6 | ns | ns |
| 2 | 6.79 | 7.01 | 0.88 | 0.33 | 7 | ns | na |
| 3 | 6.91 | 6.73 | 0.59 | 0.22 | 7 | ns | ns |
| 4 | 6.67 | 6.55 | 0.58 | 0.22 | 7 | ns | ns |

| **Variable: Cart.Bd** (mm) | | | | Levene: ns Overall p value: ns | | | |
| --- | --- | --- | --- | --- | --- | --- | --- |
| **Group** | **Mean** | **Median** | **SD** | **SEM** | **n** | **S-W** | **vs 2** |
| 1 | 7.03 | 6.73 | 0.90 | 0.37 | 6 | ns | ns |
| 2 | 6.14 | 6.19 | 0.74 | 0.28 | 7 | ns | na |
| 3 | 6.28 | 6.09 | 0.57 | 0.22 | 7 | ns | ns |
| 4 | 6.12 | 6.18 | 0.54 | 0.20 | 7 | ns | ns |

| **Variable: Cart.Wi** (µm) | | | | Levene: ns Overall p value: ns | | | |
| --- | --- | --- | --- | --- | --- | --- | --- |
| **Group** | **Mean** | **Median** | **SD** | **SEM** | **n** | **S-W** | **vs 2** |
| 1 | 222.82 | 222.52 | 26.05 | 10.63 | 6 | ns | ns |
| 2 | 274.49 | 269.08 | 43.72 | 16.53 | 7 | ns | na |
| 3 | 223.23 | 234.59 | 60.07 | 22.70 | 7 | ns | ns |
| 4 | 222.96 | 218.64 | 35.71 | 13.50 | 7 | ns | ns |

| **Variable: SCB.Wi** (µm) | | | | Levene: ns Overall p value: ns | | | |
| --- | --- | --- | --- | --- | --- | --- | --- |
| **Group** | **Mean** | **Median** | **SD** | **SEM** | **n** | **S-W** | **vs 2** |
| 1 | 488.24 | 506.03 | 65.74 | 26.84 | 6 | ns | ns |
| 2 | 600.56 | 589.33 | 101.38 | 38.32 | 7 | ns | na |
| 3 | 694.43 | 608.27 | 177.17 | 66.96 | 7 | ns | ns |
| 4 | 570.45 | 510.39 | 126.53 | 47.82 | 7 | ns | ns |

Table S2h. Histomorphometry Data Summary – Level 2 – MTP

| **Variable: BV/TV** (%) | | | | Levene: ns Overall p value: ns | | | |
| --- | --- | --- | --- | --- | --- | --- | --- |
| **Group** | **Mean** | **Median** | **SD** | **SEM** | **n** | **S-W** | **vs 2** |
| 1 | 37.17 | 40.39 | 10.12 | 4.13 | 6 | ns | ns |
| 2 | 36.81 | 38.31 | 8.92 | 3.37 | 7 | ns | na |
| 3 | 39.18 | 39.13 | 8.84 | 3.34 | 7 | ns | ns |
| 4 | 33.87 | 33.99 | 4.86 | 1.84 | 7 | ns | ns |

| **Variable: Tb.Th** (µm) | | | | Levene: ns  Overall p value: ns | | | |
| --- | --- | --- | --- | --- | --- | --- | --- |
| **Group** | **Mean** | **Median** | **SD** | **SEM** | **n** | **S-W** | **vs 2** |
| 1 | 211.66 | 206.74 | 27.28 | 11.14 | 6 | ns | ns |
| 2 | 159.60 | 157.10 | 47.16 | 17.83 | 7 | ns | na |
| 3 | 166.79 | 176.63 | 43.21 | 16.33 | 7 | ns | ns |
| 4 | 160.02 | 151.52 | 26.94 | 10.18 | 7 | ns | ns |

| **Variable: Tb.Sp** (µm) | | | | Levene: ns Overall p value: ns | | | |
| --- | --- | --- | --- | --- | --- | --- | --- |
| **Group** | **Mean** | **Median** | **SD** | **SEM** | **n** | **S-W** | **vs 2** |
| 1 | 403.66 | 315.98 | 217.96 | 88.98 | 6 | ns | ns |
| 2 | 270.55 | 253.01 | 35.56 | 13.44 | 7 | ns | na |
| 3 | 257.90 | 251.22 | 41.11 | 15.54 | 7 | ns | ns |
| 4 | 317.92 | 313.76 | 66.55 | 25.15 | 7 | ns | ns |

| **Variable: Tb.N** (1/mm) | | | | Levene: ns Overall p value: <.05 | | | |
| --- | --- | --- | --- | --- | --- | --- | --- |
| **Group** | **Mean** | **Median** | **SD** | **SEM** | **n** | **S-W** | **vs 2** |
| 1 | 1.78 | 1.89 | 0.51 | 0.21 | 6 | ns | **<.05** |
| 2 | 2.34 | 2.40 | 0.20 | 0.07 | 7 | ns | na |
| 3 | 2.37 | 2.27 | 0.21 | 0.08 | 7 | ns | ns |
| 4 | 2.15 | 1.97 | 0.44 | 0.17 | 7 | ns | ns |

| **Variable: Ophte.Ar** (mm2) | | | | Levene: ns Overall p value: <.05 | | | |
| --- | --- | --- | --- | --- | --- | --- | --- |
| **Group** | **Mean** | **Median** | **SD** | **SEM** | **n** | **S-W** | **vs 2** |
| 1 | 0.00 | 0.00 | 0.00 | 0.00 | 6 | ns | **<.05** |
| 2 | 4.17 | 2.02 | 3.85 | 1.45 | 7 | <.05 | na |
| 3 | 5.73 | 3.43 | 4.61 | 1.74 | 7 | ns | ns |
| 4 | 10.32 | 6.59 | 9.69 | 3.66 | 7 | ns | ns |

Table S2h. Histomorphometry Data Summary – Level 2 – MTP (continued)

| **Variable: Cart.Ar** (mm2) | | | | Levene: ns Overall p value: ns | | | |
| --- | --- | --- | --- | --- | --- | --- | --- |
| **Group** | **Mean** | **Median** | **SD** | **SEM** | **n** | **S-W** | **vs 2** |
| 1 | 1.16 | 0.86 | 0.71 | 0.29 | 6 | ns | ns |
| 2 | 1.09 | 1.02 | 0.50 | 0.19 | 7 | ns | na |
| 3 | 0.74 | 0.67 | 0.33 | 0.12 | 7 | ns | ns |
| 4 | 1.78 | 0.71 | 1.97 | 0.74 | 7 | ns | ns |

| **Variable: Cart.Pm** (mm) | | | | Levene: ns  Overall p value: ns | | | |
| --- | --- | --- | --- | --- | --- | --- | --- |
| **Group** | **Mean** | **Median** | **SD** | **SEM** | **n** | **S-W** | **vs 2** |
| 1 | 3.19 | 3.00 | 0.91 | 0.37 | 6 | ns | ns |
| 2 | 2.88 | 2.77 | 0.99 | 0.38 | 7 | ns | na |
| 3 | 2.43 | 2.27 | 0.59 | 0.22 | 7 | ns | ns |
| 4 | 2.70 | 2.14 | 2.28 | 0.86 | 7 | ns | ns |

| **Variable: Cart.Bd** (mm) | | | | Levene: ns Overall p value: ns | | | |
| --- | --- | --- | --- | --- | --- | --- | --- |
| **Group** | **Mean** | **Median** | **SD** | **SEM** | **n** | **S-W** | **vs 2** |
| 1 | 3.19 | 3.02 | 0.91 | 0.37 | 6 | ns | ns |
| 2 | 2.84 | 2.76 | 0.90 | 0.34 | 7 | ns | na |
| 3 | 2.32 | 2.31 | 0.39 | 0.15 | 7 | ns | ns |
| 4 | 2.50 | 2.18 | 1.90 | 0.72 | 7 | ns | ns |

| **Variable: Cart.Wi** (µm) | | | | Levene: ns Overall p value: ns | | | |
| --- | --- | --- | --- | --- | --- | --- | --- |
| **Group** | **Mean** | **Median** | **SD** | **SEM** | **n** | **S-W** | **vs 2** |
| 1 | 345.70 | 359.79 | 136.09 | 55.56 | 6 | ns | ns |
| 2 | 410.66 | 371.74 | 151.30 | 57.19 | 7 | ns | na |
| 3 | 312.01 | 270.73 | 108.52 | 41.01 | 7 | <.05 | ns |
| 4 | 282.89 | 257.34 | 220.01 | 83.15 | 7 | ns | ns |

| **Variable: SCB.Wi** (µm) | | | | Levene: ns Overall p value: ns | | | |
| --- | --- | --- | --- | --- | --- | --- | --- |
| **Group** | **Mean** | **Median** | **SD** | **SEM** | **n** | **S-W** | **vs 2** |
| 1 | 866.92 | 851.68 | 131.49 | 53.68 | 6 | ns | ns |
| 2 | 733.06 | 768.61 | 269.24 | 101.76 | 7 | ns | na |
| 3 | 747.49 | 739.43 | 209.72 | 79.26 | 7 | ns | ns |
| 4 | 575.52 | 691.04 | 319.18 | 120.64 | 7 | ns | ns |

**Rabbit anterior cruciate ligament transection model – Mankin scores**

The results of the Mankin scores are listed in Tables S2i-k. Analysis was performed on the tibial plateaus and femoral condyles at two levels (Level 1 and 2) for each:

- Medial tibial plateau (MTP)
- Lateral tibial plateau (LTP)
- Medial femoral condyle (MFC)
- Lateral femoral condyle (LFC)
- Tibial plateau (TP)
- Femoral condyle (FC)

The results are listed per group according to the following:

Group 1) sham + vehicle; Group 2) ACLT + vehicle; Group 3) ACLT + MIV-711, 30 µmol/kg (low dose) and Group 4) ACLT + MIV-711, 100 µmol/kg (high dose).

The statistical analyses in Tables S2i-k refer to the Kruskal-Wallis test.

Table S2i. Total Mankin scores – Level 1

| **Variable: Level 1_MTP** | | | | Overall p value: ns | | |
| --- | --- | --- | --- | --- | --- | --- |
| **Group** | **Mean** | **Median** | **SD** | **SEM** | **n** | **vs 2** |
| 1 | 6.33 | 7.00 | 1.86 | 0.76 | 6 | ns |
| 2 | 7.29 | 6.00 | 2.63 | 0.99 | 7 | na |
| 3 | 9.86 | 9.00 | 1.95 | 0.74 | 7 | ns |
| 4 | 9.29 | 8.00 | 3.77 | 1.43 | 7 | ns |

| **Variable: Level 1_LTP** | | | | Overall p value: ns | | |
| --- | --- | --- | --- | --- | --- | --- |
| **Group** | **Mean** | **Median** | **SD** | **SEM** | **n** | **vs 2** |
| 1 | 4.33 | 4.50 | 1.63 | 0.67 | 6 | ns |
| 2 | 5.71 | 5.00 | 1.25 | 0.47 | 7 | na |
| 3 | 5.57 | 4.00 | 3.05 | 1.15 | 7 | ns |
| 4 | 6.29 | 7.00 | 2.29 | 0.87 | 7 | ns |

| **Variable: Level 1_MFC** | | | | Overall p value: <.05 | | |
| --- | --- | --- | --- | --- | --- | --- |
| **Group** | **Mean** | **Median** | **SD** | **SEM** | **n** | **vs 2** |
| 1 | 3.33 | 3.50 | 2.07 | 0.84 | 6 | ns |
| 2 | 11.86 | 10.00 | 6.82 | 2.58 | 7 | na |
| 3 | 20.14 | 23.00 | 6.28 | 2.37 | 7 | ns |
| 4 | 14.86 | 15.00 | 8.36 | 3.16 | 7 | ns |

| **Variable: Level 1_LFC** | | | | Overall p value: <.05 | | |
| --- | --- | --- | --- | --- | --- | --- |
| **Group** | **Mean** | **Median** | **SD** | **SEM** | **n** | **vs 2** |
| 1 | 2.50 | 2.00 | 1.22 | 0.50 | 6 | ns |
| 2 | 8.00 | 7.00 | 1.83 | 0.69 | 7 | na |
| 3 | 12.00 | 10.00 | 3.92 | 1.48 | 7 | ns |
| 4 | 10.57 | 10.00 | 4.86 | 1.84 | 7 | ns |

| **Variable: Level 1_TP** | | | | Overall p value: ns | | |
| --- | --- | --- | --- | --- | --- | --- |
| **Group** | **Mean** | **Median** | **SD** | **SEM** | **n** | **vs 2** |
| 1 | 10.67 | 11.00 | 2.16 | 0.88 | 6 | ns |
| 2 | 13.00 | 13.00 | 3.06 | 1.15 | 7 | na |
| 3 | 15.43 | 16.00 | 3.31 | 1.25 | 7 | ns |
| 4 | 15.57 | 16.00 | 5.44 | 2.06 | 7 | ns |

| **Variable: Level 1_FC** | | | | Overall p value: <.05 | | |
| --- | --- | --- | --- | --- | --- | --- |
| **Group** | **Mean** | **Median** | **SD** | **SEM** | **n** | **vs 2** |
| 1 | 5.83 | 5.50 | 2.79 | 1.14 | 6 | ns |
| 2 | 19.86 | 18.00 | 7.49 | 2.83 | 7 | na |
| 3 | 32.14 | 32.00 | 7.60 | 2.87 | 7 | ns |
| 4 | 25.43 | 29.00 | 11.70 | 4.42 | 7 | ns |

Table S2j. Total Mankin scores – Level 2

| **Variable: Level 2_MTP** | | | | Overall p value: ns | | |
| --- | --- | --- | --- | --- | --- | --- |
| **Group** | **Mean** | **Median** | **SD** | **SEM** | **n** | **vs 2** |
| 1 | 5.50 | 5.00 | 0.84 | 0.34 | 6 | ns |
| 2 | 9.00 | 7.00 | 4.36 | 1.65 | 7 | na |
| 3 | 6.86 | 6.00 | 2.61 | 0.99 | 7 | ns |
| 4 | 8.14 | 7.00 | 4.30 | 1.62 | 7 | ns |

| **Variable: Level 2_LTP** | | | | Overall p value: ns | | |
| --- | --- | --- | --- | --- | --- | --- |
| **Group** | **Mean** | **Median** | **SD** | **SEM** | **n** | **vs 2** |
| 1 | 6.00 | 6.50 | 1.26 | 0.52 | 6 | ns |
| 2 | 6.00 | 6.00 | 3.27 | 1.23 | 7 | na |
| 3 | 4.14 | 4.00 | 2.34 | 0.88 | 7 | ns |
| 4 | 6.43 | 6.00 | 3.10 | 1.17 | 7 | ns |

| **Variable: Level 2_MFC** | | | | Overall p value: <.05 | | |
| --- | --- | --- | --- | --- | --- | --- |
| **Group** | **Mean** | **Median** | **SD** | **SEM** | **n** | **vs 2** |
| 1 | 3.17 | 3.00 | 0.41 | 0.17 | 6 | ns |
| 2 | 10.29 | 12.00 | 4.23 | 1.60 | 7 | na |
| 3 | 17.57 | 20.00 | 6.95 | 2.63 | 7 | ns |
| 4 | 13.71 | 13.00 | 6.52 | 2.47 | 7 | ns |

| **Variable: Level 2_LFC** | | | | Overall p value: ns | | |
| --- | --- | --- | --- | --- | --- | --- |
| **Group** | **Mean** | **Median** | **SD** | **SEM** | **n** | **vs 2** |
| 1 | 3.83 | 3.00 | 2.04 | 0.83 | 6 | ns |
| 2 | 9.00 | 7.00 | 4.51 | 1.70 | 7 | na |
| 3 | 9.14 | 7.00 | 5.08 | 1.92 | 7 | ns |
| 4 | 10.57 | 10.00 | 3.74 | 1.41 | 7 | ns |

| **Variable: Level 2_TP** | | | | Overall p value: ns | | |
| --- | --- | --- | --- | --- | --- | --- |
| **Group** | **Mean** | **Median** | **SD** | **SEM** | **n** | **vs 2** |
| 1 | 11.50 | 12.00 | 1.22 | 0.50 | 6 | ns |
| 2 | 15.00 | 12.00 | 6.51 | 2.46 | 7 | na |
| 3 | 11.00 | 12.00 | 3.87 | 1.46 | 7 | ns |
| 4 | 14.57 | 14.00 | 4.58 | 1.73 | 7 | ns |

| **Variable: Level 2_FC** | | | | Overall p value: <.05 | | |
| --- | --- | --- | --- | --- | --- | --- |
| **Group** | **Mean** | **Median** | **SD** | **SEM** | **n** | **vs 2** |
| 1 | 7.00 | 6.00 | 2.00 | 0.82 | 6 | ns |
| 2 | 19.29 | 19.00 | 5.35 | 2.02 | 7 | na |
| 3 | 26.71 | 26.00 | 10.27 | 3.88 | 7 | ns |
| 4 | 24.29 | 23.00 | 8.62 | 3.26 | 7 | ns |

Table S2k. Total Mankin scores – Level 1+2

| **Variable: Level 1+2_MTP** | | | | Overall p value: <.05 | | |
| --- | --- | --- | --- | --- | --- | --- |
| **Group** | **Mean** | **Median** | **SD** | **SEM** | **n** | **vs 2** |
| 1 | 11.83 | 12.00 | 1.17 | 0.48 | 6 | ns |
| 2 | 16.29 | 17.00 | 4.96 | 1.87 | 7 | na |
| 3 | 16.71 | 16.00 | 2.75 | 1.04 | 7 | ns |
| 4 | 17.43 | 14.00 | 7.00 | 2.64 | 7 | ns |

| **Variable: Level 1+2_LTP** | | | | Overall p value: ns | | |
| --- | --- | --- | --- | --- | --- | --- |
| **Group** | **Mean** | **Median** | **SD** | **SEM** | **n** | **vs 2** |
| 1 | 10.33 | 9.50 | 1.75 | 0.71 | 6 | ns |
| 2 | 11.71 | 10.00 | 3.30 | 1.25 | 7 | na |
| 3 | 9.71 | 10.00 | 1.89 | 0.71 | 7 | ns |
| 4 | 12.71 | 12.00 | 2.75 | 1.04 | 7 | ns |

| **Variable: Level 1+2_MFC** | | | | Overall p value: <.05 | | |
| --- | --- | --- | --- | --- | --- | --- |
| **Group** | **Mean** | **Median** | **SD** | **SEM** | **n** | **vs 2** |
| 1 | 6.50 | 7.00 | 2.17 | 0.89 | 6 | ns |
| 2 | 22.14 | 22.00 | 10.14 | 3.83 | 7 | na |
| 3 | 37.71 | 43.00 | 10.98 | 4.15 | 7 | ns |
| 4 | 28.57 | 27.00 | 14.60 | 5.52 | 7 | ns |

| **Variable: Level 1+2_LFC** | | | | Overall p value: <.05 | | |
| --- | --- | --- | --- | --- | --- | --- |
| **Group** | **Mean** | **Median** | **SD** | **SEM** | **n** | **vs 2** |
| 1 | 6.33 | 5.00 | 2.16 | 0.88 | 6 | ns |
| 2 | 17.00 | 15.00 | 6.16 | 2.33 | 7 | na |
| 3 | 21.14 | 17.00 | 8.80 | 3.33 | 7 | ns |
| 4 | 21.14 | 21.00 | 5.84 | 2.21 | 7 | ns |

| **Variable: Level 1+2_TP** | | | | Overall p value: <.05 | | |
| --- | --- | --- | --- | --- | --- | --- |
| **Group** | **Mean** | **Median** | **SD** | **SEM** | **n** | **vs 2** |
| 1 | 22.17 | 22.00 | 2.04 | 0.83 | 6 | ns |
| 2 | 28.00 | 28.00 | 5.69 | 2.15 | 7 | na |
| 3 | 26.43 | 26.00 | 2.64 | 1.00 | 7 | ns |
| 4 | 30.14 | 27.00 | 7.58 | 2.87 | 7 | ns |

| **Variable: Level 1+2_FC** | | | | Overall p value: <.05 | | |
| --- | --- | --- | --- | --- | --- | --- |
| **Group** | **Mean** | **Median** | **SD** | **SEM** | **n** | **vs 2** |
| 1 | 12.83 | 13.50 | 3.19 | 1.30 | 6 | ns |
| 2 | 39.14 | 37.00 | 12.21 | 4.62 | 7 | na |
| 3 | 58.86 | 58.00 | 16.77 | 6.34 | 7 | ns |
| 4 | 49.71 | 51.00 | 18.65 | 7.05 | 7 | ns |
