# Supplementary material for: The selective cathepsin K inhibitor MIV-711 attenuates joint pathology in experimental animal models of osteoarthritis
Source: J Transl Med. 2018 Mar 9;16:56. doi: 10.1186/s12967-018-1425-7 (PMC5845353; doi:10.1186/s12967-018-1425-7)
Supplement: Supplementary file 4 — Additional file 4: Table S3. Dog partial medial meniscectomy model - summary of microscopic pathology. [file 12967_2018_1425_MOESM4_ESM.docx]

**Additional file 4: Table S3.**

**Dog partial medial meniscectomy model - Microscopic scoring**

**Table S3a. Summary of Microscopic Pathology**

| **Treatment Group** | **Tibia** | | | | | | | **Femur** | | | | | | | **Osteophyte** | | **Bone** | **Total Joint** |
| --- | --- | --- | --- | --- | --- | --- | --- | --- | --- | --- | --- | --- | --- | --- | --- | --- | --- | --- |
| **Animal #** | **Cartilage Degeneration** | | | | | | **Mean DRAMC#** | **Cartilage Degeneration** | | | | | | **Mean DRAMC#** | **Score##** | **Measure (µm)** |  |  |
| **1** | **Score*** | | | | **Width (µm)** | |  | **Score*** | | | | **Width (µm)** | |  |  |  |  |  |
| **MIV-711** | **Mean** | **Level 1** | **Level 2** | **Level 3** | **Total**** | **Sig***** |  | **Mean** | **Level 1** | **Level 2** | **Level 3** | **Total**** | **Sig***** |  |  |  | **Score^**  **###** | **CD Score** |
| 1 (1413237) | 1.17 | 1.00 | 1.25 | 1.25 | 7245 | 2163 | 0.14 | 0.67 | 0.75 | 0.50 | 0.75 | 3990 | 815 | 0.08 | 0.00 0 | | 1 | 1.83 |
| 2 (1407725) | 1.42 | 1.00 | 1.75 | 1.50 | 8400 | 4515 | 0.20 | 1.58 | 1.00 | 1.75 | 2.00 | 5880 | 3360 | 0.31 | 0.00 0 | | 4 | 3.00 |
| 3 (1412257) | 0.92 | 1.50 | 1.25 | 0.00 | 8400 | 1785 | 0.13 | 0.33 | 0.50 | 0.25 | 0.25 | 2730 | 0 | 0.03 | 0.00 0 | | 3 | 1.25 |
| 4 (1407563) | 0.67 | 0.50 | 1.00 | 0.50 | 7770 | 1470 | 0.09 | 0.67 | 0.50 | 0.50 | 1.00 | 3675 | 1068 | 0.12 | 0.00 0 | | 2 | 1.33 |
| 5 (1409868) | 0.58 | 0.25 | 1.00 | 0.50 | 6405 | 1638 | 0.07 | 0.50 | 0.50 | 0.50 | 0.50 | 3885 | 0 | 0.04 | 0.00 0 | | 5 | 1.08 |
| 6 (1410513) | 1.00 | 0.25 | 1.25 | 1.50 | 6510 | 1890 | 0.13 | 0.67 | 0.50 | 1.00 | 0.50 | 4200 | 735 | 0.06 | 0.00 0 | | 3 | 1.67 |
| 7 (1413288) | 0.92 | 0.00 | 1.25 | 1.50 | 8190 | 2310 | 0.13 | 0.83 | 0.50 | 1.00 | 1.00 | 4410 | 1260 | 0.07 | 0.00 0 | | 4 | 1.75 |
| 8 (1410173) | 0.67 | 0.00 | 1.00 | 1.00 | 7560 | 1470 | 0.11 | 0.08 | 0.25 | 0.00 | 0.00 | 630 | 0 | 0.00 | 0.00 0 | | 3 | 0.75 |
| 16 (1421230) | 1.08 | 0.25 | 1.75 | 1.25 | 8400 | 2520 | 0.17 | 1.33 | 0.75 | 1.75 | 1.50 | 5040 | 3465 | 0.28 | 0.00 0 | | 5 | 2.42 |
| 17 (1406087) | 0.75 | 0.50 | 1.00 | 0.75 | 7980 | 2205 | 0.09 | 0.58 | 0.75 | 0.50 | 0.50 | 3780 | 200 | 0.04 | 0.00 0 | | 3 | 1.33 |
| 18 (1416449) | 0.92 | 0.50 | 1.50 | 0.75 | 6195 | 1155 | 0.15 | 0.58 | 0.50 | 0.75 | 0.50 | 3885 | 968 | 0.07 | 0.00 0 | | 2 | 1.50 |
| 19 (1416937) | 0.92 | 0.50 | 1.25 | 1.00 | 6510 | 2520 | 0.11 | 0.33 | 0.50 | 0.25 | 0.25 | 2310 | 420 | 0.03 | 0.00 0 | | 3 | 1.25 |
| 20 (1410939) | 1.08 | 1.25 | 1.25 | 0.75 | 6930 | 3150 | 0.16 | 0.25 | 0.25 | 0.00 | 0.50 | 2310 | 0 | 0.01 | 0.00 0 | | 2 | 1.33 |
| 21 (1405421) | 1.17 | 0.75 | 1.25 | 1.50 | 6615 | 1575 | 0.17 | 0.83 | 0.75 | 0.50 | 1.25 | 4200 | 1890 | 0.07 | 0.00 0 | | 3 | 2.00 |
| 22 (1421922) | 0.42 | 0.75 | 0.50 | 0.00 | 6930 | 1218 | 0.07 | 0.67 | 0.50 | 0.50 | 1.00 | 3990 | 733 | 0.04 | 0.00 0 | | 3 | 1.08 |
| **Mean** | **0.91** | **0.60** | **1.22** | **0.92** | **7336.0** | **2105.6** | **0.13** | **0.66** | **0.57** | **0.65** | **0.77** | **3661.0** | **994.3** | **0.084** | **0.00 0.00** | | **3.07** | **1.57** |
| **SE** | **0.07** | **0.11** | **0.08** | **0.13** | **209.0** | **223.0** | **0.01** | **0.10** | **0.05** | **0.14** | **0.14** | **323.7** | **290.0** | **0.024** | **0.00 0.00** | | **0.28** | **0.15** |
| **t-test to Vehicle** | **NS** | **Sig** | **NS** | **NS** | **1.00** | **0.74** | **0.25** | **NS** | **NS** | **NS** | **NS** | **0.19** | **0.72** | **0.79** | **NS #DIV/0!** | | **NS** | **NS** |
| **% inhibition** | **9%** | **41%** | **9%** | **-38%** | **0%** | **5%** | **17%** | **7%** | **13%** | **0%** | **8%** | **13%** | **11%** | **-9%** | **#DIV/0! #DIV/0!** | | **4%** | **8%** |
| **Treatment Group**  **Animal # 2**  **Vehicle** | **Tibia** | | | | | | | **Femur** | | | | | | | **Osteophyte** | | **Bone Score** | **Total Joint**  **CD Score** |
|  | **Cartilage Degeneration** | | | | | | **Mean DRAMC#** | **Cartilage Degeneration** | | | | | | **Mean DRAMC#** | **Score##** | **Measure (µm)** |  |  |
|  | **Score*** | | | | **Width (µm)** | |  | **Score*** | | | | **Width (µm)** | |  |  |  |  |  |
|  | **Mean** | **Level 1** | **Level 2** | **Level 3** | **Total**** | **Sig***** |  | **Mean** | **Level 1** | **Level 2** | **Level 3** | **Total**** | **Sig***** |  |  |  |  |  |
| 9 (1405706) | 0.58 | 0.00 | 1.25 | 0.50 | 5880 | 525 | 0.06 | 0.67 | 0.25 | 0.75 | 1.00 | 3990 | 1097 | 0.05 | 0.00 0 | | 2 | 1.25 |
| 10 (1422546) | 0.92 | 1.00 | 1.75 | 0.00 | 7035 | 1995 | 0.16 | 0.83 | 0.75 | 0.75 | 1.00 | 4620 | 1602 | 0.10 | 0.00 0 | | 4 | 1.75 |
| 11 (1411935) | 0.83 | 0.50 | 1.50 | 0.50 | 7350 | 1260 | 0.09 | 0.83 | 0.75 | 0.75 | 1.00 | 4935 | 1345 | 0.06 | 0.00 0 | | 4 | 1.67 |
| 12 (1418638) | 1.67 | 2.00 | 1.75 | 1.25 | 7455 | 3675 | 0.31 | 0.33 | 0.25 | 0.25 | 0.50 | 2520 | 210 | 0.02 | 0.00 0 | | 3 | 2.00 |
| 13 (1414594) | 1.08 | 1.00 | 0.75 | 1.50 | 7140 | 1890 | 0.13 | 0.50 | 0.25 | 0.25 | 1.00 | 3570 | 735 | 0.06 | 0.00 0 | | 3 | 1.58 |
| 14 (1427106) | 1.00 | 1.25 | 1.00 | 0.75 | 7140 | 2520 | 0.13 | 0.92 | 0.75 | 1.25 | 0.75 | 4935 | 1527 | 0.13 | 0.00 0 | | 3 | 1.92 |
| 15 (1408683) | 1.08 | 1.25 | 1.75 | 0.25 | 7980 | 3465 | 0.17 | 0.83 | 0.75 | 0.75 | 1.00 | 4410 | 1680 | 0.11 | 0.00 0 | | 5 | 1.92 |
| 23 (1416210) | 1.42 | 1.75 | 1.75 | 0.75 | 9030 | 3570 | 0.24 | 1.00 | 0.75 | 1.00 | 1.25 | 5670 | 1575 | 0.13 | 0.00 0 | | 4 | 2.42 |
| 24 (1413687) | 0.58 | 0.75 | 0.75 | 0.25 | 7350 | 630 | 0.06 | 0.25 | 0.25 | 0.25 | 0.25 | 2100 | 0 | 0.02 | 0.00 0 | | 4 | 0.83 |
| 25 (1425082) | 1.33 | 1.25 | 1.00 | 1.75 | 8295 | 3255 | 0.22 | 0.92 | 1.25 | 0.75 | 0.75 | 4410 | 1575 | 0.10 | 0.00 0 | | 4 | 2.25 |
| 26 (1425880) | 0.67 | 0.50 | 1.00 | 0.50 | 7035 | 1050 | 0.06 | 0.75 | 0.50 | 0.75 | 1.00 | 5355 | 210 | 0.06 | 0.00 0 | | 2 | 1.42 |
| 27 (1422482) | 1.00 | 0.75 | 1.25 | 1.00 | 6930 | 3150 | 0.20 | 0.75 | 0.75 | 0.50 | 1.00 | 4200 | 1680 | 0.08 | 0.00 0 | | 3 | 1.75 |
| 28 (1410352) | 1.17 | 1.50 | 1.50 | 0.50 | 7980 | 3150 | 0.20 | 0.58 | 0.75 | 0.50 | 0.50 | 3780 | 735 | 0.05 | 0.00 0 | | 3 | 1.75 |
| 29 (1416759) | 0.92 | 1.00 | 1.75 | 0.00 | 6300 | 2205 | 0.15 | 0.67 | 1.00 | 0.50 | 0.50 | 3780 | 1050 | 0.07 | 0.00 0 | | 1 | 1.58 |
| 30 (1416686) | 0.83 | 0.75 | 1.25 | 0.50 | 7140 | 1050 | 0.11 | 0.83 | 0.75 | 0.75 | 1.00 | 4830 | 1680 | 0.12 | 0.00 0 | | 3 | 1.67 |
| **Mean** | **1.01** | **1.02** | **1.33** | **0.67** | **7336.0** | **2226.0** | **0.15** | **0.71** | **0.65** | **0.65** | **0.83** | **4207.0** | **1113.3** | **0.077** | **0.00 0.00** | | **3.20** | **1.72** |
| **SE** | **0.08** | **0.13** | **0.10** | **0.13** | **198.7** | **289.5** | **0.02** | **0.06** | **0.08** | **0.07** | **0.07** | **251.3** | **154.5** | **0.009** | **0.00 0.00** | | **0.26** | **0.10** |
| **% inhibition** | **0%** | **0%** | **0%** | **0%** | **0%** | **0%** | **0%** | **0%** | **0%** | **0%** | **0%** | **0%** | **0%** | **0%** | **#DIV/0! #DIV/0!** | | **0%** | **0%** |
| *Cartilage degeneration score=depth (1-5) Mean for each of 4 quadrants  **Width of any cartilage lesion (mean of 3 anterior to posterior sections)  ***Width of cartilage degeneration with score of 1 or greater  #Depth Ratio Any Matrix Change=Sum of Mean Lesion depth in µm vs depths to tidemark over 4 sites on the tibia or femur (mean of the 4 quadrants)  ##Osteophyte scores 1=up to 2000 µm, 2=2001-3000 µm, 3=greater than 3001 µm (mean of 3 sections)  ^Scoring of sclerosis of subchondral bone | | | | | | | | | | | | | | | | | | |

**Table S3b. Cartilage degeneration by zones**

| **1**  **MIV-711** | **Tibia** | | | | | | **Femur** | | | | | |
| --- | --- | --- | --- | --- | --- | --- | --- | --- | --- | --- | --- | --- |
|  | **Cartilage Degeneration Score** | | | | | | **Cartilage Degeneration Score** | | | | | |
|  | **Zone 1** | **Zone 2** | | **Zone 3** | | **Zone 4** | **Zone 1** | **Zone 2** | | **Zone 3** | | **Zone 4** |
| 1 (1413237) | 0.33 | 2.33 |  | | 1.00 | 1.00 | 1.33 | 1.00 |  | | 0.33 | 0.00 |
| 2 (1407725) | 0.67 | 2.33 |  | | 1.67 | 1.00 | 2.00 | 2.33 |  | | 1.67 | 0.33 |
| 3 (1412257) | 0.00 | 2.00 |  | | 1.00 | 0.67 | 1.00 | 0.33 |  | | 0.00 | 0.00 |
| 4 (1407563) | 0.00 | 1.67 |  | | 0.33 | 0.67 | 1.33 | 1.33 |  | | 0.00 | 0.00 |
| 5 (1409868) | 0.33 | 1.33 |  | | 0.67 | 0.00 | 1.00 | 1.00 |  | | 0.00 | 0.00 |
| 6 (1410513) | 0.33 | 1.67 |  | | 1.33 | 0.67 | 1.00 | 1.33 |  | | 0.33 | 0.00 |
| 7 (1413288) | 0.67 | 1.33 |  | | 1.00 | 0.67 | 1.00 | 1.67 |  | | 0.33 | 0.33 |
| 8 (1410173) | 0.00 | 1.33 |  | | 1.00 | 0.33 | 0.33 | 0.00 |  | | 0.00 | 0.00 |
| 16 (1421230) | 0.67 | 1.67 |  | | 1.33 | 0.67 | 2.00 | 2.00 |  | | 1.33 | 0.00 |
| 17 (1406087) | 0.33 | 1.67 |  | | 1.00 | 0.00 | 1.00 | 1.00 |  | | 0.33 | 0.00 |
| 18 (1416449) | 0.67 | 1.33 |  | | 1.00 | 0.67 | 1.00 | 1.33 |  | | 0.00 | 0.00 |
| 19 (1416937) | 0.00 | 2.00 |  | | 1.33 | 0.33 | 1.33 | 0.00 |  | | 0.00 | 0.00 |
| 20 (1410939) | 0.00 | 2.00 |  | | 1.67 | 0.67 | 0.67 | 0.33 |  | | 0.00 | 0.00 |
| 21 (1405421) | 1.00 | 2.33 |  | | 0.67 | 0.67 | 1.00 | 1.67 |  | | 0.33 | 0.33 |
| 22 (1421922) | 0.00 | 1.33 |  | | 0.33 | 0.00 | 1.33 | 1.00 |  | | 0.33 | 0.00 |
| **Mean** | **0.33** | **1.76** |  | | **1.02** | **0.53** | **1.16** | **1.09** |  | | **0.33** | **0.07** |
| **SE** | **0.09** | **0.10** |  | | **0.11** | **0.08** | **0.11** | **0.18** |  | | **0.13** | **0.04** |
| **t-test to Vehicle** | **NS** | **NS** |  | | **NS** | **NS** | **NS** | **NS** |  | | **NS** | **NS** |
| **% inhibition** | **48%** | **0%** |  | | **8%** | **-4%** | **-6%** | **6%** |  | | **32%** | **40%** |
| **2**  **Vehicle** | **Tibia** | | | | | | **Femur** | | | | | |
|  | **Cartilage Degeneration Score** | | | | | | **Cartilage Degeneration Score** | | | | | |
|  | **Zone 1** | **Zone 2** | | **Zone 3** | | **Zone 4** | **Zone 1** | **Zone 2** | | **Zone 3** | | **Zone 4** |
| 9 (1405706) | 0.67 | 1.00 | 0.33 | | | 0.33 | 1.67 | 0.67 | 0.00 | | | 0.33 |
| 10 (1422546) | 0.33 | 1.67 | 1.00 | | | 0.67 | 1.67 | 1.33 | 0.33 | | | 0.00 |
| 11 (1411935) | 0.33 | 1.33 | 1.33 | | | 0.33 | 1.33 | 1.00 | 1.00 | | | 0.00 |
| 12 (1418638) | 2.00 | 2.67 | 1.67 | | | 0.33 | 0.67 | 0.67 | 0.00 | | | 0.00 |
| 13 (1414594) | 0.33 | 2.33 | 1.00 | | | 0.67 | 1.00 | 0.67 | 0.00 | | | 0.33 |
| 14 (1427106) | 0.00 | 2.00 | 1.00 | | | 1.00 | 1.33 | 1.33 | 0.67 | | | 0.33 |
| 15 (1408683) | 0.33 | 2.00 | 1.33 | | | 0.67 | 1.00 | 1.67 | 0.67 | | | 0.00 |
| 23 (1416210) | 1.33 | 2.33 | 1.33 | | | 0.67 | 1.00 | 1.67 | 1.00 | | | 0.33 |
| 24 (1413687) | 0.33 | 1.00 | 0.33 | | | 0.67 | 0.67 | 0.33 | 0.00 | | | 0.00 |
| 25 (1425082) | 1.33 | 2.33 | 1.33 | | | 0.33 | 1.00 | 1.67 | 1.00 | | | 0.00 |
| 26 (1425880) | 0.00 | 0.67 | 1.00 | | | 1.00 | 1.00 | 1.00 | 0.67 | | | 0.33 |
| 27 (1422482) | 0.33 | 2.00 | 1.67 | | | 0.00 | 1.00 | 1.33 | 0.67 | | | 0.00 |
| 28 (1410352) | 1.00 | 2.67 | 1.00 | | | 0.00 | 0.67 | 1.33 | 0.33 | | | 0.00 |
| 29 (1416759) | 0.33 | 1.33 | 1.33 | | | 0.67 | 1.00 | 1.33 | 0.33 | | | 0.00 |
| 30 (1416686) | 1.00 | 1.00 | 1.00 | | | 0.33 | 1.33 | 1.33 | 0.67 | | | 0.00 |
| **Mean** | **0.64** | **1.76** | **1.11** | | | **0.51** | **1.09** | **1.16** | **0.49** | | | **0.11** |
| **SE** | **0.15** | **0.17** | **0.10** | | | **0.08** | **0.08** | **0.11** | **0.10** | | | **0.04** |
| **% inhibition** | **0%** | **0%** | **0%** | | | **0%** | **0%** | **0%** | **0%** | | | **0%** |

**Table S3c. Depth Ratio Any Matrix Change by Zones**

| **1**  **MIV-711** | **Tibia** | | |  | | **Femur** | | |  | |
| --- | --- | --- | --- | --- | --- | --- | --- | --- | --- | --- |
|  | **Depth Ratio Any Matrix Change** | | | |  | **Depth Ratio Any Matrix Change** | | | |  |
|  | **Zone 1** | **Zone 2** | **Zone 3** | **Zone 4** | **Mean** | **Zone 1** | **Zone 2** | **Zone 3** | **Zone 4** | **Mean** |
| 1 (1413237) | 0.11 | 0.39 | 0.04 | 0.04 | 0.14 | 0.22 | 0.09 | 0.01 | 0.00 | 0.08 |
| 2 (1407725) | 0.13 | 0.47 | 0.19 | 0.03 | 0.20 | 0.50 | 0.48 | 0.21 | 0.06 | 0.31 |
| 3 (1412257) | 0.00 | 0.33 | 0.15 | 0.04 | 0.13 | 0.06 | 0.05 | 0.00 | 0.00 | 0.03 |
| 4 (1407563) | 0.00 | 0.30 | 0.03 | 0.03 | 0.09 | 0.23 | 0.24 | 0.02 | 0.00 | 0.12 |
| 5 (1409868) | 0.03 | 0.19 | 0.05 | 0.02 | 0.07 | 0.09 | 0.08 | 0.00 | 0.00 | 0.04 |
| 6 (1410513) | 0.06 | 0.28 | 0.14 | 0.05 | 0.13 | 0.08 | 0.13 | 0.04 | 0.00 | 0.06 |
| 7 (1413288) | 0.08 | 0.28 | 0.11 | 0.05 | 0.13 | 0.07 | 0.16 | 0.03 | 0.01 | 0.07 |
| 8 (1410173) | 0.01 | 0.23 | 0.14 | 0.04 | 0.11 | 0.01 | 0.00 | 0.00 | 0.00 | 0.00 |
| 16 (1421230) | 0.09 | 0.35 | 0.18 | 0.05 | 0.17 | 0.42 | 0.36 | 0.33 | 0.00 | 0.28 |
| 17 (1406087) | 0.03 | 0.24 | 0.08 | 0.00 | 0.09 | 0.07 | 0.08 | 0.01 | 0.00 | 0.04 |
| 18 (1416449) | 0.13 | 0.27 | 0.15 | 0.04 | 0.15 | 0.19 | 0.10 | 0.00 | 0.00 | 0.07 |
| 19 (1416937) | 0.00 | 0.36 | 0.07 | 0.00 | 0.11 | 0.10 | 0.00 | 0.00 | 0.00 | 0.03 |
| 20 (1410939) | 0.00 | 0.35 | 0.24 | 0.07 | 0.16 | 0.01 | 0.01 | 0.01 | 0.00 | 0.01 |
| 21 (1405421) | 0.15 | 0.39 | 0.08 | 0.06 | 0.17 | 0.09 | 0.16 | 0.02 | 0.02 | 0.07 |
| 22 (1421922) | 0.00 | 0.22 | 0.06 | 0.01 | 0.07 | 0.09 | 0.06 | 0.01 | 0.00 | 0.04 |
| **Mean** | **0.05** | **0.31** | **0.11** | **0.04** | **0.13** | **0.15** | **0.13** | **0.05** | **0.01** | **0.08** |
| **SE** | **0.01** | **0.02** | **0.02** | **0.01** | **0.01** | **0.04** | **0.03** | **0.02** | **0.00** | **0.02** |
| **t-test to Vehicle** | **0.07** | **0.88** | **0.21** | **0.38** | **0.25** | **0.29** | **0.73** | **0.85** | **0.51** | **0.79** |
| **% inhibition** | **55%** | **-2%** | **23%** | **19%** | **17%** | **-39%** | **10%** | **-12%** | **44%** | **-9%** |
| **2**  **Vehicle** | **Tibia** | | | | | **Femur** | | | | |
|  | **Depth Ratio Any Matrix Change** | | | | | **Depth Ratio Any Matrix Change** | | | | |
|  | **Zone 1** | **Zone 2** | **Zone 3** | **Zone 4** | **Mean** | **Zone 1** | **Zone 2** | **Zone 3** | **Zone 4** | **Mean** |
| 9 (1405706) | 0.05 | 0.14 | 0.02 | 0.01 | 0.06 | 0.11 | 0.06 | 0.00 | 0.02 | 0.05 |
| 10 (1422546) | 0.06 | 0.32 | 0.18 | 0.08 | 0.16 | 0.22 | 0.15 | 0.01 | 0.00 | 0.10 |
| 11 (1411935) | 0.03 | 0.19 | 0.13 | 0.02 | 0.09 | 0.14 | 0.07 | 0.04 | 0.00 | 0.06 |
| 12 (1418638) | 0.42 | 0.58 | 0.25 | 0.00 | 0.31 | 0.06 | 0.03 | 0.00 | 0.00 | 0.02 |
| 13 (1414594) | 0.02 | 0.32 | 0.12 | 0.05 | 0.13 | 0.13 | 0.10 | 0.00 | 0.01 | 0.06 |
| 14 (1427106) | 0.03 | 0.31 | 0.10 | 0.09 | 0.13 | 0.12 | 0.26 | 0.05 | 0.08 | 0.13 |
| 15 (1408683) | 0.09 | 0.31 | 0.23 | 0.06 | 0.17 | 0.13 | 0.27 | 0.04 | 0.00 | 0.11 |
| 23 (1416210) | 0.30 | 0.46 | 0.14 | 0.06 | 0.24 | 0.07 | 0.29 | 0.12 | 0.03 | 0.13 |
| 24 (1413687) | 0.04 | 0.14 | 0.02 | 0.05 | 0.06 | 0.06 | 0.03 | 0.00 | 0.00 | 0.02 |
| 25 (1425082) | 0.22 | 0.44 | 0.18 | 0.04 | 0.22 | 0.14 | 0.23 | 0.04 | 0.00 | 0.10 |
| 26 (1425880) | 0.00 | 0.08 | 0.09 | 0.09 | 0.06 | 0.07 | 0.15 | 0.02 | 0.01 | 0.06 |
| 27 (1422482) | 0.09 | 0.43 | 0.27 | 0.00 | 0.20 | 0.12 | 0.15 | 0.03 | 0.00 | 0.08 |
| 28 (1410352) | 0.26 | 0.39 | 0.15 | 0.01 | 0.20 | 0.06 | 0.13 | 0.02 | 0.00 | 0.05 |
| 29 (1416759) | 0.07 | 0.24 | 0.24 | 0.06 | 0.15 | 0.07 | 0.10 | 0.10 | 0.00 | 0.07 |
| 30 (1416686) | 0.13 | 0.19 | 0.10 | 0.03 | 0.11 | 0.12 | 0.21 | 0.13 | 0.01 | 0.12 |
| **Mean** | **0.12** | **0.30** | **0.15** | **0.04** | **0.15** | **0.11** | **0.15** | **0.04** | **0.01** | **0.08** |
| **SE** | **0.03** | **0.04** | **0.02** | **0.01** | **0.02** | **0.01** | **0.02** | **0.01** | **0.01** | **0.01** |
| **% inhibition** | **0%** | **0%** | **0%** | **0%** | **0%** | **0%** | **0%** | **0%** | **0%** | **0%** |

**Table S3d. Summary of individual animal collagen degeneration – percentage of total width**

| **1**  **MIV-711** | **Normal** | **Total** | **Severe** | **Marked Moderate** | | **Mild** | **Minimal** | **Severe+ Marked+**  **Moderate+ Mild** | **Severe+ Marked+**  **Moderate** | **Mild+ Minimal** | **Severe+ Marked** | **Moderate+ Mild+**  **Minimal** |
| --- | --- | --- | --- | --- | --- | --- | --- | --- | --- | --- | --- | --- |
| 1 (1413237) | 62% | 38% | 0% | 0% | 0% | 25% | 8% | 25% | 0% | 34% | 0% | 34% |
| 2 (1407725) | 42% | 58% | 0% | 0% | 0% | 30% | 27% | 30% | 0% | 58% | 0% | 58% |
| 3 (1412257) | 71% | 29% | 0% | 0% | 4% | 13% | 12% | 17% | 4% | 25% | 0% | 29% |
| 4 (1407563) | 82% | 18% | 0% | 0% | 2% | 14% | 2% | 16% | 2% | 16% | 0% | 18% |
| 5 (1409868) | 72% | 28% | 0% | 0% | 0% | 8% | 20% | 8% | 0% | 28% | 0% | 28% |
| 6 (1410513) | 74% | 26% | 0% | 0% | 3% | 16% | 7% | 19% | 3% | 23% | 0% | 26% |
| 7 (1413288) | 70% | 30% | 0% | 0% | 0% | 16% | 14% | 16% | 0% | 30% | 0% | 30% |
| 8 (1410173) | 81% | 19% | 0% | 0% | 1% | 10% | 9% | 11% | 1% | 18% | 0% | 19% |
| 16 (1421230) | 69% | 31% | 0% | 0% | 0% | 26% | 5% | 26% | 0% | 31% | 0% | 31% |
| 17 (1406087) | 77% | 23% | 0% | 0% | 0% | 11% | 12% | 11% | 0% | 23% | 0% | 23% |
| 18 (1416449) | 76% | 24% | 0% | 0% | 10% | 4% | 10% | 14% | 10% | 14% | 0% | 24% |
| 19 (1416937) | 65% | 35% | 0% | 0% | 0% | 31% | 4% | 31% | 0% | 35% | 0% | 35% |
| 20 (1410939) | 61% | 39% | 0% | 0% | 0% | 27% | 12% | 27% | 0% | 39% | 0% | 39% |
| 21 (1405421) | 73% | 27% | 0% | 0% | 7% | 13% | 7% | 20% | 7% | 20% | 0% | 27% |
| 22 (1421922) | 76% | 24% | 0% | 0% | 0% | 13% | 12% | 13% | 0% | 24% | 0% | 24% |
| **Mean** | **70%** | **30%** | **0%** | **0%** | **2%** | **17%** | **11%** | **19%** | **2%** | **28%** | **0%** | **30%** |
| **SE** | **3%** | **3%** | **0%** | **0%** | **1%** | **2%** | **2%** | **2%** | **1%** | **3%** | **0%** | **2%** |
| **T-test to Vehicle** | **0.36** | **0.36** | **0.33** | **0.33** | **0.71** | **0.92** | **0.13** | **0.97** | **0.71** | **0.39** | **0.33** | **0.32** |
| **% inhibition** | **-6%** | **12%** | **100%** | **100%** | **21%** | **-2%** | **28%** | **1%** | **21%** | **12%** | **100%** | **12%** |

**Table S3d. Summary of individual animal collagen degeneration – percentage of total width (continued)**

| **2**  **Vehicle** | **Normal** | **Total** | **Severe** | **Marked Moderate** | | **Mild** | **Minimal** | **Severe+ Marked+**  **Moderate+ Mild** | **Severe+ Marked+**  **Moderate** | **Mild+ Minimal** | **Severe+ Marked** | **Moderate+ Mild+**  **Minimal** |
| --- | --- | --- | --- | --- | --- | --- | --- | --- | --- | --- | --- | --- |
| 9 (1405706) | 84% | 16% | 0% | 0% | 0% | 4% | 12% | 4% | 0% | 16% | 0% | 16% |
| 10 (1422546) | 67% | 33% | 0% | 0% | 2% | 16% | 14% | 18% | 2% | 31% | 0% | 33% |
| 11 (1411935) | 61% | 39% | 0% | 0% | 0% | 9% | 30% | 9% | 0% | 39% | 0% | 39% |
| 12 (1418638) | 55% | 45% | 0% | 0% | 8% | 29% | 8% | 37% | 8% | 37% | 0% | 45% |
| 13 (1414594) | 76% | 25% | 0% | 0% | 0% | 18% | 6% | 18% | 0% | 24% | 0% | 24% |
| 14 (1427106) | 69% | 31% | 0% | 0% | 0% | 15% | 16% | 15% | 0% | 31% | 0% | 31% |
| 15 (1408683) | 44% | 56% | 0% | 0% | 0% | 34% | 23% | 34% | 0% | 56% | 0% | 56% |
| 23 (1416210) | 57% | 43% | 0% | 0% | 13% | 20% | 10% | 33% | 13% | 30% | 0% | 43% |
| 24 (1413687) | 89% | 11% | 0% | 0% | 0% | 2% | 8% | 2% | 0% | 11% | 0% | 11% |
| 25 (1425082) | 59% | 41% | 0% | 0% | 0% | 29% | 12% | 29% | 0% | 41% | 0% | 41% |
| 26 (1425880) | 86% | 14% | 0% | 0% | 0% | 7% | 7% | 7% | 0% | 14% | 0% | 14% |
| 27 (1422482) | 55% | 45% | 0% | 0% | 0% | 32% | 14% | 32% | 0% | 45% | 0% | 45% |
| 28 (1410352) | 63% | 37% | 0% | 0% | 7% | 22% | 8% | 29% | 7% | 30% | 0% | 37% |
| 29 (1416759) | 57% | 43% | 0% | 0% | 6% | 8% | 29% | 13% | 6% | 37% | 0% | 43% |
| 30 (1416686) | 71% | 29% | 0% | 0% | 0% | 7% | 22% | 7% | 0% | 29% | 0% | 29% |
| **Mean** | **66%** | **34%** | **0%** | **0%** | **2%** | **17%** | **15%** | **19%** | **2%** | **32%** | **0%** | **34%** |
| **SE** | **3%** | **3%** | **0%** | **0%** | **1%** | **3%** | **2%** | **3%** | **1%** | **3%** | **0%** | **3%** |
| **% inhibition** | **0%** | **0%** | **0%** | **0%** | **0%** | **0%** | **0%** | **0%** | **0%** | **0%** | **0%** | **0%** |

**Table S3e. Cartilage Area**

| 1  MIV-711 | Total  Area | Non-viable  Area | Viable  Area | % Viable  Area | No  Matrix | Any  Matrix | % Any Matrix  Area |
| --- | --- | --- | --- | --- | --- | --- | --- |
|  | 611181 | 19981 | 591200 | 97% | 3398 | 607783 | 99% |
| 1 (1413237) |  |  |  |  |  |  |  |
| L2 | 782085 | 64430 | 717655 | 92% | 19782 | 762302 | 97% |
| L3 | 658261 | 66664 | 591597 | 90% | 9255 | 649006 | 99% |
| 2 (1407725) | 736682 | 21737 | 714946 | 97% | 5521 | 731162 | 99% |
| L2 | 705629 | 124827 | 580802 | 82% | 36398 | 669231 | 95% |
| L3 | 524778 | 160376 | 364401 | 69% | 74917 | 449860 | 86% |
| 3 (1412257) | 669782 | 148310 | 521472 | 78% | 16687 | 653095 | 98% |
| L2 | 827272 | 138166 | 689106 | 83% | 22376 | 804896 | 97% |
| L3 | 770329 | 49380 | 720949 | 94% | 0 | 770329 | 100% |
| 4 (1407563) | 798502 | 49785 | 748717 | 94% | 0 | 798502 | 100% |
| L2 | 792520 | 113022 | 679498 | 86% | 72849 | 719671 | 91% |
| L3 | 743041 | 79094 | 663947 | 89% | 35286 | 707754 | 95% |
| 5 (1409868) | 734449 | 30301 | 704148 | 96% | 0 | 734449 | 100% |
| L2 | 696264 | 71387 | 624877 | 90% | 10069 | 686195 | 99% |
| L3 | 582619 | 62461 | 520159 | 89% | 13370 | 569250 | 98% |
| 6 (1410513) | 799030 | 32212 | 766817 | 96% | 0 | 799030 | 100% |
| L2 | 948121 | 134023 | 814098 | 86% | 30958 | 917163 | 97% |
| L3 | 788568 | 106417 | 682151 | 87% | 38486 | 750083 | 95% |
| 7 (1413288) | 851036 | 51874 | 799162 | 94% | 0 | 851036 | 100% |
| L2 | 977811 | 144385 | 833426 | 85% | 42175 | 935637 | 96% |
| L3 | 851112 | 199617 | 651496 | 77% | 64572 | 786540 | 92% |
| 8 (1410173) | 633774 | 0 | 633774 | 100% | 0 | 633774 | 100% |
| L2 | 741602 | 50106 | 691496 | 93% | 7735 | 733867 | 99% |
| L3 | 648237 | 95887 | 552350 | 85% | 34468 | 613770 | 95% |
| 16 (1421230) | 889139 | 0 | 889139 | 100% | 0 | 889139 | 100% |
| L2 | 713888 | 194974 | 518914 | 73% | 63561 | 650327 | 91% |
| L3 | 607499 | 121893 | 485606 | 80% | 45923 | 561576 | 92% |
| 17 (1406087) | 745615 | 57582 | 688034 | 92% | 0 | 745615 | 100% |
| L2 | 810293 | 116623 | 693670 | 86% | 9513 | 800781 | 99% |
| L3 | 693717 | 87690 | 606027 | 87% | 31323 | 662395 | 95% |
| 18 (1416449) | 747693 | 46159 | 701533 | 94% | 0 | 747693 | 100% |
| L2 | 783632 | 133386 | 650246 | 83% | 69998 | 713635 | 91% |
| L3 | 647149 | 71588 | 575561 | 89% | 26966 | 620182 | 96% |
| 19 (1416937) | 781668 | 100395 | 681273 | 87% | 28606 | 753062 | 96% |
| L2 | 716486 | 110452 | 606034 | 85% | 57324 | 659162 | 92% |
| L3 | 738557 | 104354 | 634203 | 86% | 61664 | 676893 | 92% |
| 20 (1410939) | 787965 | 158606 | 629359 | 80% | 17049 | 770916 | 98% |
| L2 | 752378 | 92475 | 659903 | 88% | 23154 | 729224 | 97% |
| L3 | 734139 | 44950 | 689188 | 94% | 21659 | 712480 | 97% |
| 21 (1405421) | 519856 | 57340 | 462515 | 89% | 11145 | 508710 | 98% |
| L2 | 714579 | 103613 | 610965 | 86% | 52179 | 662399 | 93% |
| L3 | 606467 | 97451 | 509016 | 84% | 47556 | 558912 | 92% |
| 22 (1421922) | 740398 | 70431 | 669967 | 90% | 31032 | 709366 | 96% |
| L2 | 691126 | 32073 | 659053 | 95% | 10807 | 680319 | 98% |
| L3 | 655599 | 0 | 655599 | 100% | 0 | 655599 | 100% |
| Mean |  |  |  | **88%** |  |  | **96%** |
| SE |  |  |  | **1%** |  |  | **1%** |
| t-test to Vehicle |  |  |  | **0.54** |  |  | **0.60** |
| Percent Inhibition |  |  |  | **8%** |  |  | **-11%** |

**Table S3e. Cartilage Area (continued)**

| 2  Vehicle | Total  Area | Non-viable  Area | Viable  Area | % Viable  Area | No  Matrix | Any  Matrix | % Any Matrix  Area |
| --- | --- | --- | --- | --- | --- | --- | --- |
|  | 581443 | 7416 | 574027 | 99% | 0 | 581443 | 100% |
| 9 (1405706) |  |  |  |  |  |  |  |
| L2 | 837567 | 42071 | 795497 | 95% | 4153 | 833415 | 100% |
| L3 | 687657 | 25734 | 661924 | 96% | 9359 | 678299 | 99% |
| 10 (1422546) | 769143 | 111526 | 657617 | 85% | 25947 | 743197 | 97% |
| L2 | 790357 | 172785 | 617572 | 78% | 57421 | 732937 | 93% |
| L3 | 576259 | 0 | 576259 | 100% | 0 | 576259 | 100% |
| 11 (1411935) | 796978 | 33509 | 763469 | 96% | 0 | 796978 | 100% |
| L2 | 738419 | 92695 | 645724 | 87% | 9323 | 729096 | 99% |
| L3 | 690388 | 16507 | 673881 | 98% | 0 | 690388 | 100% |
| 12 (1418638) | 688606 | 207430 | 481176 | 70% | 53542 | 635064 | 92% |
| L2 | 692182 | 256666 | 435516 | 63% | 57134 | 635047 | 92% |
| L3 | 612234 | 85400 | 526834 | 86% | 29761 | 582473 | 95% |
| 13 (1414594) | 749001 | 63410 | 685592 | 92% | 14053 | 734948 | 98% |
| L2 | 779035 | 35613 | 743422 | 95% | 13005 | 766029 | 98% |
| L3 | 658493 | 82650 | 575842 | 87% | 34281 | 624212 | 95% |
| 14 (1427106) | 853985 | 81600 | 772385 | 90% | 21469 | 832515 | 97% |
| L2 | 828630 | 68187 | 760442 | 92% | 22468 | 806162 | 97% |
| L3 | 698545 | 54285 | 644259 | 92% | 7795 | 690750 | 99% |
| 15 (1408683) | 798843 | 146739 | 652104 | 82% | 32780 | 766062 | 96% |
| L2 | 715128 | 199981 | 515147 | 72% | 19901 | 695227 | 97% |
| L3 | 724825 | 41370 | 683455 | 94% | 3862 | 720963 | 99% |
| 23 (1416210) | 860722 | 166529 | 694193 | 81% | 43147 | 817575 | 95% |
| L2 | 771716 | 207828 | 563888 | 73% | 64070 | 707646 | 92% |
| L3 | 696846 | 71787 | 625059 | 90% | 27875 | 668971 | 96% |
| 24 (1413687) | 634527 | 16048 | 618478 | 97% | 0 | 634527 | 100% |
| L2 | 846593 | 66375 | 780218 | 92% | 14581 | 832011 | 98% |
| L3 | 764636 | 16077 | 748559 | 98% | 0 | 764636 | 100% |
| 25 (1425082) | 760627 | 127317 | 633310 | 83% | 27761 | 732866 | 96% |
| L2 | 814212 | 123798 | 690414 | 85% | 31647 | 782565 | 96% |
| L3 | 703057 | 166429 | 536628 | 76% | 65125 | 637932 | 91% |
| 26 (1425880) | 928478 | 26962 | 901517 | 97% | 0 | 928478 | 100% |
| L2 | 805582 | 81559 | 724023 | 90% | 28637 | 776945 | 96% |
| L3 | 914378 | 92371 | 822007 | 90% | 4508 | 909870 | 100% |
| 27 (1422482) | 646895 | 91010 | 555885 | 86% | 14820 | 632075 | 98% |
| L2 | 761735 | 178812 | 582922 | 77% | 55336 | 706398 | 93% |
| L3 | 582892 | 106013 | 476879 | 82% | 42809 | 540083 | 93% |
| 28 (1410352) | 783284 | 135745 | 647539 | 83% | 55071 | 728213 | 93% |
| L2 | 701465 | 182104 | 519361 | 74% | 84636 | 616829 | 88% |
| L3 | 734877 | 53864 | 681013 | 93% | 19678 | 715199 | 97% |
| 29 (1416759) | 912201 | 110156 | 802044 | 88% | 15447 | 896753 | 98% |
| L2 | 830156 | 262056 | 568100 | 68% | 45208 | 784948 | 95% |
| L3 | 611798 | 0 | 611798 | 100% | 0 | 611798 | 100% |
| 30 (1416686) | 983363 | 106439 | 876924 | 89% | 3209 | 980154 | 100% |
| L2 | 1045000 | 79655 | 965345 | 92% | 13938 | 1031062 | 99% |
| L3 | 609214 | 37028 | 572186 | 94% | 0 | 609214 | 100% |
| Mean |  |  |  | **87%** |  |  | **97%** |
| SE |  |  |  | **1%** |  |  | **0%** |
| Percent Inhibition |  |  |  | **0%** |  |  | **0%** |

**Table S3f. Bone Area Percentage**

|  | **Percent Length of no Bone** | | | | | | | | |
| --- | --- | --- | --- | --- | --- | --- | --- | --- | --- |
| **1**  **MIV-711** | **1** | **Site 2** | **LFC**  **3** | **Mean** | **1** | **Site 2** | **MFC**  **3** | **Mean** | **Overall Mean** |
| 1 (1413237) | 46% | 59% | 60% | 55% | 57% | 51% | 49% | 52% | 54% |
| 2 (1407725) | 57% | 59% | 47% | 54% | 44% | 27% | 44% | 38% | 46% |
| 3 (1412257) | 66% | 45% | 46% | 52% | 48% | 52% | 36% | 46% | 49% |
| 4 (1407563) | 53% | 60% | 56% | 56% | 51% | 57% | 41% | 50% | 53% |
| 5 (1409868) | 66% | 72% | 71% | 70% | 21% | 26% | 21% | 22% | 46% |
| 6 (1410513) | 67% | 60% | 52% | 60% | 38% | 36% | 40% | 38% | 49% |
| 7 (1413288) | 58% | 50% | 42% | 50% | 47% | 32% | 34% | 38% | 44% |
| 8 (1410173) | 64% | 62% | 48% | 58% | 50% | 42% | 33% | 42% | 50% |
| 16 (1421230) | 49% | 55% | 49% | 51% | 24% | 21% | 21% | 22% | 37% |
| 17 (1406087) | 53% | 63% | 64% | 60% | 35% | 39% | 36% | 37% | 48% |
| 18 (1416449) | 63% | 66% | 60% | 63% | 52% | 46% | 51% | 50% | 56% |
| 19 (1416937) | 54% | 57% | 58% | 56% | 38% | 38% | 24% | 33% | 45% |
| 20 (1410939) | 70% | 51% | 45% | 55% | 52% | 52% | 51% | 51% | 53% |
| 21 (1405421) | 57% | 57% | 49% | 54% | 36% | 37% | 32% | 35% | 45% |
| 22 (1421922) | 61% | 54% | 59% | 58% | 36% | 34% | 22% | 30% | 44% |
| **Mean** | **59%** | **58%** | **54%** | **57%** | **42%** | **39%** | **36%** | **39%** | **48%** |
| **SE** | **2%** | **2%** | **2%** | **1%** | **3%** | **3%** | **3%** | **2%** | **1%** |
| **T-test to Vehicle** | **0.44** | **0.70** | **0.20** | **0.69** | **0.22** | **0.96** | **0.13** | **0.32** | **0.47** |
| **% inhibition** | **-4%** | **2%** | **6%** | **1%** | **-12%** | **1%** | **-21%** | **-10%** | **-3%** |
| **2**  **Vehicle** | **1** | **Site 2** | **LFC**  **3** | **Mean** | **1** | **Site 2** | **MFC**  **3** | **Mean** | **Overall Mean** |
| 9 (1405706) | 40% | 64% | 51% | 51% | 40% | 65% | 53% | 53% | 52% |
| 10 (1422546) | 61% | 59% | 60% | 60% | 25% | 27% | 27% | 26% | 43% |
| 11 (1411935) | 61% | 62% | 62% | 62% | 28% | 28% | 28% | 28% | 45% |
| 12 (1418638) | 57% | 54% | 57% | 56% | 37% | 41% | 18% | 32% | 44% |
| 13 (1414594) | 51% | 55% | 56% | 54% | 50% | 44% | 41% | 45% | 49% |
| 14 (1427106) | 57% | 41% | 41% | 46% | 38% | 43% | 25% | 35% | 41% |
| 15 (1408683) | 43% | 49% | 62% | 51% | 25% | 31% | 15% | 24% | 38% |
| 23 (1416210) | 58% | 73% | 55% | 62% | 31% | 32% | 20% | 27% | 45% |
| 24 (1413687) | 69% | 65% | 59% | 64% | 39% | 33% | 16% | 29% | 47% |
| 25 (1425082) | 55% | 55% | 58% | 56% | 38% | 24% | 22% | 28% | 42% |
| 26 (1425880) | 65% | 65% | 56% | 62% | 36% | 54% | 30% | 40% | 51% |
| 27 (1422482) | 53% | 59% | 52% | 55% | 28% | 35% | 27% | 30% | 42% |
| 28 (1410352) | 55% | 60% | 65% | 60% | 45% | 49% | 30% | 42% | 51% |
| 29 (1416759) | 52% | 65% | 66% | 61% | 51% | 47% | 52% | 50% | 56% |
| 30 (1416686) | 74% | 60% | 63% | 65% | 51% | 40% | 36% | 42% | 54% |
| **Mean** | **57%** | **59%** | **57%** | **58%** | **37%** | **40%** | **29%** | **35%** | **47%** |
| **SE** | **2%** | **2%** | **2%** | **1%** | **2%** | **3%** | **3%** | **2%** | **1%** |
| **% inhibition** | **0%** | **0%** | **0%** | **0%** | **0%** | **0%** | **0%** | **0%** | **0%** |
